# Means of enhancing bone fracture healing: optimal cell source, isolation methods and acoustic stimulation

**DOI:** 10.1186/s12896-016-0318-1

**Published:** 2016-12-12

**Authors:** Corina Adriana Ghebes, Maaike Vera Jasmijn Braham, Adelgunde Veronica Clemens Maria Zeegers, Auke Jan Sijbe Renard, Hugo Fernandes, Daniel B F Saris

**Affiliations:** 1 MIRA Institute for Biomedical Technology and Technical Medicine, University of Twente, Drienerlolaan 5, 7522NB Enschede, The Netherlands; 2 Department of Orthopedic Surgery, Medisch Spectrum Twente Hospital, Postbus 50 000, 7500KA Enschede, The Netherlands; 3 Department of Orthopaedics, University Medical Center Utrecht, Heidelberglaan 100, 3584 CX Utrecht, The Netherlands; 4 Center for Neuroscience and Cell Biology (CNC), Stem Cells and Drug Screening Lab, University of Coimbra, Largo Marques de Pombal, 3004-517 Coimbra, Portugal; 5 Faculty of Science and Technology, Institute Technical Medicine, University of Twente, P.O. Box 217, 7500 AE Enschede, The Netherlands

**Keywords:** Bone marrow, hMSCs, Acoustic stimulation, Cell priming

## Abstract

**Background:**

The human body has an extensive capacity to regenerate bone tissue after trauma. However large defects such as long bone fractures of the lower limbs cannot be restored without intervention and often lead to nonunion. Therefore, the aim of the present study was to assess the pool and biological functions of human mesenchymal stromal cells (hMSCs) isolated from different bone marrow locations of the lower limbs and to identify novel strategies to prime the cells prior to their use in bone fracture healing. Following, bone marrow from the ilium, proximal femur, distal femur and proximal tibia was aspirated and the hMSCs isolated. Bone marrow type, volume, number of mononuclear cells/hMSCs and their self-renewal, multilineage potential, extracellular matrix (ECM) production and surface marker profiling were analyzed. Additionally, the cells were primed to accelerate bone fracture healing either by using acoustic stimulation or varying the initial hMSCs isolation conditions.

**Results:**

We found that the more proximal the bone marrow aspiration location, the larger the bone marrow volume was, the higher the content in mononuclear cells/hMSCs and the higher the self-renewal and osteogenic differentiation potential of the isolated hMSCs were. Acoustic stimulation of bone marrow, as well as the isolation of hMSCs in the absence of fetal bovine serum, increased the osteogenic and ECM production potential of the cells, respectively.

**Conclusion:**

We showed that bone marrow properties change with the aspiration location, potentially explaining the differences in bone fracture healing between the tibia and the femur. Furthermore, we showed two new priming methods capable of enhancing bone fracture healing.

**Electronic supplementary material:**

The online version of this article (doi:10.1186/s12896-016-0318-1) contains supplementary material, which is available to authorized users.

## Background

Musculoskeletal disorders affect the body’s muscles, bones, joints, tendons, ligaments and nerves and are the leading cause of chronic disabilities in adults [1]. Significant research efforts have been undertaken during the last decades to ease this disability and improve patient’s mobility and quality of life. Bone fracture repairs have been intensively investigated at both clinical and fundamental level and still 5-10% of fractures resulted in either delayed repair (delayed union) or no repair (nonunion) [2]. At present there are two primary treatment strategies: (1) surgical intervention that implies the use of bone autograft/allografts, demineralized bone matrix or synthetic materials and (2) noninvasive treatments such as the application of acoustic energy shown to be beneficial in fracture healing [3, 4]. Nevertheless, these strategies rely on the patient’s own cells – either stem and/or committed- to induce bone regeneration, posing a challenge in situation whereas those cells are missing and/or less active. In these cases cell-based alternatives, such as the use of human mesenchymal stromal cells (hMSCs) were proposed [5].

In this study we explored the yield, proliferation, multilineage differentiation and extracellular matrix (ECM) production potential of hMSCs isolated from bone marrow (BM) aspirated from the lower limbs, such as the ilium, proximal femur, distal femur and proximal tibia. Additionally, we examined the inter- and intra-donor variation between the BM-derived hMSCs from the different locations. It has been shown that the nonunion rate in bone fracture healing (BFH) differs with regard to their location, with fractures at tibia diaphysis healing slower (nonunion rate of 18.5% [6]) than fractures in the femoral shaft (nonunion rate of 1.7% [7]). Accordingly, we hypothesize that BM located at the fracture site might play an important role in the fracture healing rate, due to differences in cell number, self-renewal-, proliferative-, ECM production- and multilineage differentiation potential.

Additionally, as cell-based therapies are already used in musculoskeletal pathologies, such as bone fracture, pseudarthrosis and osteochondral defects [8, 9], we explored the potential of priming BM-derived hMSCs towards the  osteogenic lineage in order to accelerate tissue regeneration upon reimplantation. We explored two distinct priming strategies: (1) the use of acoustic energy applied on BM and (2) varying the initial culture conditions of the isolated hMSCs.

Ultrasound has been shown to have beneficial effects on BFH showing an increase in bone formation [10, 11], however not consistently [12–14]. Moreover, a 42% acceleration in fracture healing in patients exposed to a twenty minutes daily ultrasound treatment is still not optimal [11]. Therefore, we believe that the use of acoustically stimulated BM injected at the fracture site might have a greater impact on BFH than the actual standard ultrasound treatment. Mechanical stimulation has been shown to pre-commit hMSCs towards the osteogenic lineage [15] and thus we hypothesize that acoustic energy applied directly on BM might induce the commitment of hMSCs towards osteogenesis. It is clinically feasible and simple to apply a short period of acoustic stimulation on a BM aspirate during fracture surgery after which the BM can be administrated to the fracture site either in initial surgery during high risk cases or as an adjuvant to revision surgery in case of pseudarthrosis.

Secondly, cell-based therapies often involve the in vitro expansion of cells, where the isolation procedure plays an important role in the selection of desired cell population [16–18]. The isolation of hMSCs from BM is mainly achieved by plastic adherence and it is recognized that both the number of mononuclear cells (MNCs) plated and the culture media have a strong influence on the selection of certain hMSCs populations [19]. Accordingly, we hypothesize that low MNCs seeding density might select hMSCs with higher self-renewal potential, while the use of serum free (SF) media might select a hMSCs subpopulation with enhanced potency. The phenotype of the isolated hMSCs under the aforementioned conditions were compared to a previously described isolation procedure [20].

**Fig. 1:**
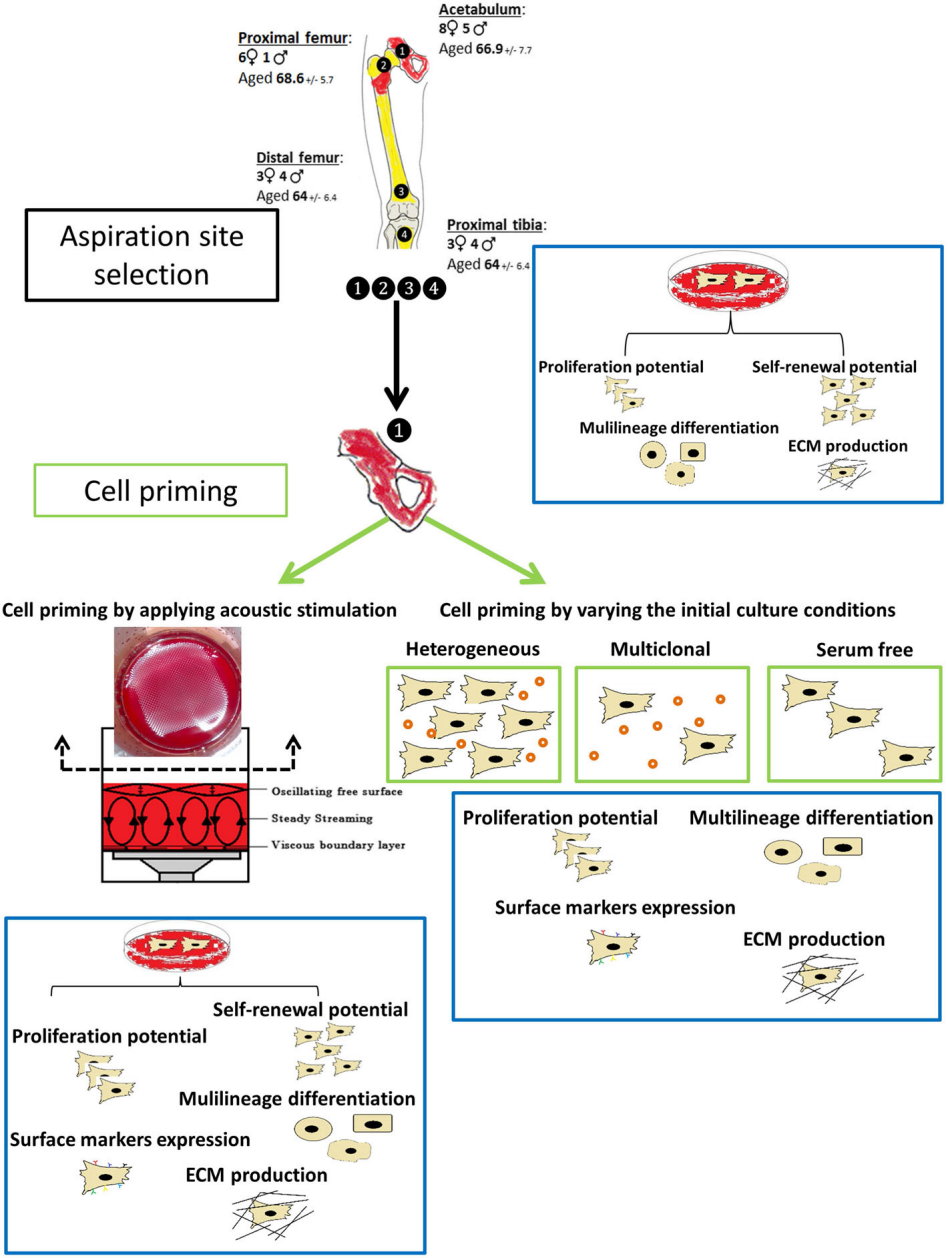
Schematic representation of the experimental design. Aspiration of BM from different locations of the lower limb extremities and selection of the optimal cell source, based on hMSC number and phenotype. In vitro priming of hMSC by use of acoustic stimulation or varying the initial culture conditions with the final aim to enhance in vivo bone fracture healing

Following, with this study we aimed to find the optimal ratio between aspirated BM volume and MNCs concentration, to explain the difference in cell phenotype between the different BM locations of the lower limb extremities and to propose new methods that could accelerate BFH. A schematic overview of the experimental design is presented in Fig. 1.

## Methods

### Aspiration of bone marrow

BM aspirates were obtained from patients undergoing total hip arthroplasty (THA) or total knee arthroplasty (TKA). An 8G Jamshidi BM needle fit with a 50-mL Luer lock syringe containing 1 mL of 1,000 U heparin per 10 mL BM was used to aspirate the BM. Subsequently, the BM was transferred to blood collection tubes (BD-367526) for the transport from the operating theatre to the laboratory. The BM was kept at ambient room temperature until processed within the same day.

BM was aspirated from four different locations: the supra acetabular sulcus (ilium) in twelve donors, the medullary cavity or lateral diaphysis of the femur (proximal femur) in seven donors, and the epiphysis or medullary cavity from the distal femur or proximal tibia in seven more donors.

### Isolation and culture of hMSCs

BM aspirate was passed through a 70 μm pore-size cell strainer to remove the presence of tissue pieces after which MNCs concentration from the ilium and proximal femur was analyzed using the Beckman coulter ACT diff 2. The number of MNCs for samples collected from the distal femur and proximal tibia was not analyzed due to technical limitations.

Based on the isolation method used, different concentrations of MNCs/cm^2^ were plated. We defined three isolation/culture conditions: heterogeneous (classical MNCs seeding density, previously described and standardized hMSCs isolation protocol within our laboratory [21]), multiclonal (low MNCs seeding density, permissive for single cell clonal expansion) and SF (high MNCs seeding density in the absence of fetal bovine serum proteins during the initial phase).

For the heterogeneous isolation condition, BM aspirate was plated at a density of 5x10^5^ MNCs/cm^2^ and cultured in growth media (GM) consisting of α-minimal essential media (*α*MEM, Life Technologies – Cat. No.: 22571-020), 10% Fetal Bovine Serum (FBS, Gibco – Cat. No.: 10270106), 0.2 mM L-ascorbic acid 2-phosphate magnesium salt (Sigma – Cat. No.: A8960), 2 mM L-glutamine (Gibco – Cat. No.: BE17-605E), 100 units/mL penicillin and 100 mg/mL streptomycin (Gibco – Cat. No.: 15140-122).

For the multiclonal isolation condition, BM aspirate was plated at a clonal density of 5x10^4^ MNCs/cm^2^ and cultured in GM.

For the SF condition, BM aspirate was plated at a cell density of 1.5x10^6^ MNC/cm^2^ in α-MEM containing no additives for the first three days.

At the fourth day, the non-adherent cell fraction was removed and the media was changed to GM for all three conditions. Hereafter, media was refreshed twice a week. At semi-confluence cells were trypsinized and used for sub-culturing or stored in liquid nitrogen for future use.

In total, BM was aspirated from 19 donors and subsequently divided between the different experiments. BM from 14 donors was used to evaluate the most convenient aspiration site location. BM was plated under heterogeneous condition, with exception of distal Femur and proximal Tibia where 2 ml BM was plated each time, as the initial amount of MNCs was unknown. BM from 11 donors was plated in heterogenous condition ﻿and﻿ used to evaluate the effects of acoustic energy stimulation and BM from 6 donors was used to evaluate the effects of varying the initial isolation conditions of the hMSCs.

Donor number, BM aspiration location, BM volume and concentration of MNCs/ml can be found in Table 1. BM was cultured at 37 °C and 5% CO_2_.

**Table 1:** BM information

Donor number	BM aspiration location	BM color	BM aspirated volume (ml)	MNC/ml (10^6^)
Donor 1	Ilium	Red	20	31.6
Donor 2	Ilium	Red	35	12.3
Donor 3	Ilium	Red	15	17.9
Donor 4	Ilium	Red	25	20.2
Donor 5	Ilium	Red	30	13.5
Donor 6	Ilium	Red	28	6.8
	Proximal Femur	Red	11.5	16.3
Donor 7	Ilium	Red	20	16.1
	Proximal Femur	Red	1	15.1
Donor 8	Ilium	Red	13	34.7
	Proximal Femur	Red	6	16.2
Donor 9	Ilium	Red	20	7.3
	Proximal Femur	Red	6.5	7.9
Donor 10	Ilium	Red	22.5	14.5
	Proximal Femur	Red	9	22.4
Donor 11	Ilium	Red	22.5	7.4
	Proximal Femur	Red	5	6.4
Donor 12	Ilium	Red	7.5	26.6
	Proximal Femur	Red	5	37
Donor 13	Distal Femur	Yellow	3.5	-
	Proximal Tibia	Yellow	2.5	-
Donor 14	Distal Femur	Yellow	5	-
	Proximal Tibia	Yellow	7.5	-
Donor 15	Distal Femur	Yellow	9	-
	Proximal Tibia	Yellow	3	-
Donor 16	Distal Femur	Yellow	2	-
	Proximal Tibia	Yellow	1.5	-
Donor 17	Distal Femur	Yellow	6	-
	Proximal Tibia	Yellow	7	-
Donor 18	Distal Femur	Yellow	8	-
	Proximal Tibia	Yellow	4.5	-
Donor 19	Distal Femur	Yellow	2	-
	Proximal Tibia	Yellow	2	-

From *left* to *right*: donor number, BM aspiration location, type, aspirated volume and concentration of MNCs/ml

### hMSCs population doubling

To assess hMSC proliferation, cells from passage 1 (P1) were seeded in GM at 5 000 cells/cm^2^ in T175 tissue culture flasks. At semi confluence the cells were trypsinized and counted. Population doubling (PD) was calculated according to the formula PD = log_2_(N_E_/N_i_), where N_E_ and N_I_ are the number of hMSCs obtained at passage 2 (P2) and P1, respectively.

### Colony forming unit and colony forming unit-osteoblast potential (mineralization)

The colony forming unit (CFU) assay was used as an indicator of self-renewal potential of the hMSCs and the CFU-osteoblast (CFU-Ob) assay was used as an indicator of their osteogenic potential. Two million MNCs were seeded in duplicate in T25 culture flasks and grown in GM for the first 7 days, followed by transition to mineralization media for further 7 days. The mineralization media consisted of GM containing 0.01 M β-glycerophosphate (BGP, Sigma – Cat. No.: G9422) and 10^-8^ M Dexamethasone (Dex, Sigma – Cat. No.: D8893). At day 14, the cultures were fixed with 10% formalin for 15 min at ambient temperature, after which alkaline phosphatase (ALP) positive colonies were stained using the Leukocyte Alkaline Phosphatase Kit -ALP (Sigma – Cat. No.: 85 L2) following the manufacturer’s instructions. Subsequently, colonies were stained using 0.5% Coomassie Brilliant Blue staining (Fluka – Cat. No.: 27815) solution for 10 minutes and images of the stained colonies were acquired using an Epson Perfection V750 PRO scanner. The total number of CFUs and ALP positive colonies was quantified using ImageJ 1.45 s software and the percentage of ALP positive CFUs calculated.

### Extracellular matrix production

hMSCs at P2 or P3 were seeded in quadruplicate at 100 000 cells/well in a 384-well plate in GM (without serum) consisting of 50 μg/mL insulin transferrin selenium-premix (Sigma – Cat. No.: I3146) and 40 μg/mL proline (Sigma – Cat. No.: P5607) and incubated for 24 h to allow cell adhesion. The next day the medium was refreshed and 10 ng/mL transforming growth factor beta 3 (R&D Systems – Cat. No.: 243-B3) and 10^-7^ M Dex was added to the wells. After seven days the formed nodules were fixed in 10% formalin for 15 min at ambient temperature and images were captured using a Nikon bright field microscope. The nodule area and the number of nodules formed were quantified using ImageJ 1.45 s software. The early cell condensation phenotype and the increase in nodule size was associated with ECM production.

### Alizarin red staining (mineralization)

hMSCs at P2 or P3 were seeded in triplicate at 50 000 cells/well in T25 and grown in control media consisting of GM containing 0.01 M BGP and in mineralization medium consisting of GM containing 0.01 M BGP and 10^-8^ M Dex. The media was refreshed twice a week. After 28 days, cells were fixed in 10% formalin for 15 min at ambient temperature and stained with 2% Alizarin red solution (Sigma – Cat. No.: A5533) for 5 min. Images were captured using a Nikon bright field microscope.

### Oil red O staining (Adipogenesis)

hMSCs at P2 or P3 were seeded in triplicate at 25 000 cells/well in 24-well plates and grown in control medium consisting of GM or adipogenic medium consisting of GM containing 0.2 mM indomethacin (Cat. No.: I7378), 0.5 mM isobutylmethylxanthine (Cat. No.: I5879), 10^-6^ M Dex and 10 μg/mL human insulin (Cat. No.: I9278), all from Sigma. The media was refreshed twice a week. After three weeks the cells were fixed with 10% formalin for 15 min at ambient temperature, after which the cell monolayer was incubated for 5 min in 60% isopropanol, and subsequently stained with Oil red O solution (3 mg/mL in 60% isopropanol, Sigma – Cat. No.: 0625). After five minutes, samples were rinsed with demineralized water and images were captured using a Nikon bright field microscope. After the imaging, Oil red O staining was extracted from the cells in 4% Nonidet P40 (Fluka, Cat. No.: 74385) in isopropanol and absorbance was measured at 540 nm (Lambda 40; Perkin Elmer). One hundred percent Oil red O was included in the calibration curve measurements, from which the percentage of Oil red O staining was calculated.

### Flow cytometry

hMSCs at P3 or P4 were expanded in T175 until they reached confluence. The cells were trypsinized and incubated for 30 min in blocking buffer consisting of 17% bovine serum albumin (Sigma – Cat. No.: F7524) in PBS followed by incubation with FITC- or PE-conjugated mouse anti-human antibodies for 30 min at 4 °C in the dark. The samples were then washed three times with a washing buffer consisting of 3% bovine serum albumin in PBS. The expression levels were analyzed using FACSAria flow cytometer (BD Bioscience). For phenotypic characterization the following antibodies were used: CD90, CD73, CD146, CD105, CD271, CD34, CD14, CD79a, HLA-DR, CD45 and IgG1 and Ig G2a as isotype controls (all from BD Pharming).

### Acoustic stimulation of bone marrow

Acoustic stimulation of BM was achieved using the bone marrow aspirate concentration device, previously described by Ridgway et al. [22]. BM was placed into the processing chamber of the device and acoustic vibration was applied using a voice-coil which produced a geometric standing waveform pattern on the BM fluid surface. Different frequencies were tested by manual adjustment using an Oscilloscope (Agilent Technologies, InfiniVision, MSO-X-3014A Mixed Signal Oscilloscope) and two frequencies, 300 Hz (48 mW/cm^2^) and 500 Hz (73 mW/cm^2^), were selected for further experimental research. The BM was processed one time for 5 and 10 min for both selected frequencies. The baseline was defined as unstimulated BM.

Following, part of the BM was plated to assess the self-renewal and proliferation potential as previously described, while the rest of the BM was plated under the heterogeneous hMSCs isolation condition in order to assess the multilineage differentiation potential, ECM production and surface markers expression of the hMSCs at later passage, as previously mentioned.

### Bone marrow viscosity

BM viscosity from 6 donors (3 donors for ilium and proximal femur and 3 donors for distal femur and proximal tibia) was measured using the Rheometer Physica MCR-301. A total of thirty different points, with an increasing shear rate from 0 to 250 L/s and periodic pause of 10 s between each point, were measured. The volume of BM used for the measurements was 350 μl per measuring cycle. All samples were measured in duplicates at ambient room temperature.

### Statistical analysis

Statistical analysis was performed using Graphpad Prism 6 software. Unpaired Student’s *t*-test and Mann-Whitney post-test was performed to compare the data when two groups were analyzed. One-way or two-way ANOVA and a Tukey or Bonferroni post-test was used to compare the data when more than two groups were analyzed. The uniform distribution of data, to test inter-donor variation, was assessed using a Chi-squared test. A P ≤ 0.05 indicates a statistical significant difference. The results are shown as mean ± standard deviation.

## Results

### Inter-donor variability in bone marrow aspirate

**Fig. 2:**
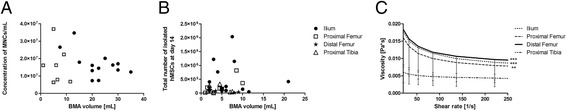
Characterization of BM aspirated (BMA) from different locations. **a** Correlation between aspirated BM volume and MNCs concentration, for the ilium (circle) and proximal femur (square). **b** Correlation between the plated BM volumes and the number of isolated hMSCs, heterogeneous isolation condition only. **c** BM viscosity curves from different aspiration locations, represented as correlation between the shear rate and the viscosity. The values represented the mean ± standard deviation of three BM donors (*n* = 3). Statistically significant differences were found with ***p < 0.001 and **p < 0.01

The volume of BM aspirated from the different locations varied significant, with larger BM volumes obtained from the ilium (22 ± 7.6 ml) than the proximal femur (6 ± 3.3 ml), distal femur (5 ± 2.9 ml) or proximal tibia (4 ± 2.6 ml). BM volumes from the ilium yielded a higher concentration of MNCs for volumes close to 10 ml (2.6 x 10^7^ MNC/ml), while volumes close and larger than 20 ml yielded a lower concentration of MNCs (1.4 x 10^7^ MNC/ml), however not statistical significant (p = 0.15). Similarly, BM aspirated from proximal femur showed higher MNC yield for volumes lower than 5 mL, 2 x 10^7^ MNC/mL versus 1.6 x 10^7^ MNC/mL, however not statistical significant (p = 0.7) (Fig. 2A and Table 1).

The concentration of hMSCs obtained at the end of the expansion phase (14 days after MNCs seeding) was on average 566 494 hMSC/ml for the ilium, 245 549 hMSC/ml for the proximal femur, 76 250 hMSC/ml for the distal femur and 122 321 hMSC/ml for the proximal tibia samples (Fig. 2B). No statistical significant differences were found between the groups, however lower p values where obtained from hMSCs isolated from proximal versus distal locations (Additional file 1: Table S1).

Macroscopically, the BM aspirated from the ilium and proximal femur was red, while BM aspirated from distal femur and proximal tibia was yellow, consistent with a higher presence of lipid droplets in the latter (Additional file 2: Figure S1). Compared with the other aspiration locations we observed a significant decrease in BM viscosity for BM aspirated from the proximal tibia (Fig. 2C). The morphological appearance of expanded hMSCs did not show any visible differences between the different BM aspiration locations (data not shown).

### Effect of aspiration location on biological characteristics of hMSCs

**Fig. 3:**
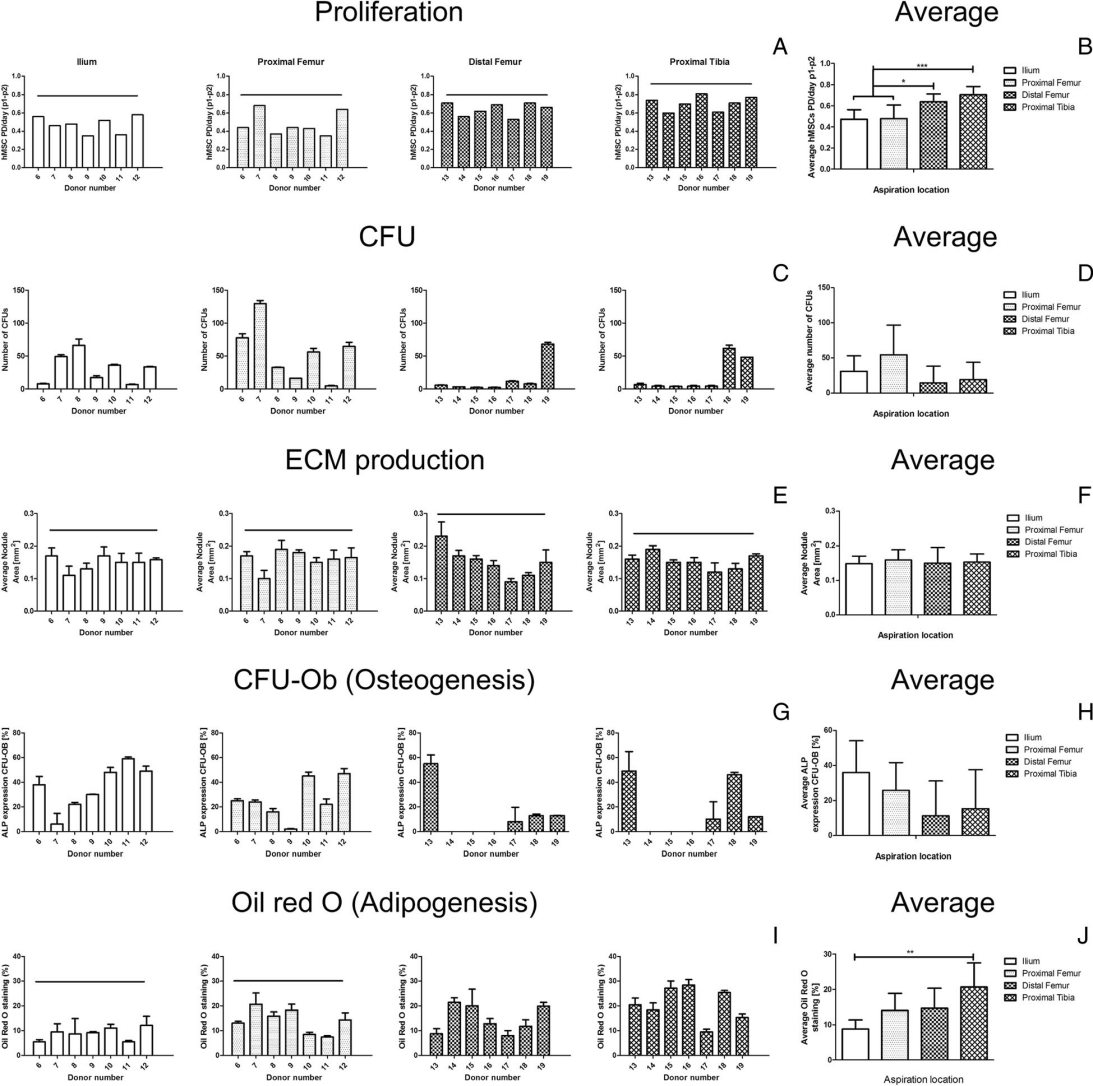
Biological characterization of hMSCs isolated from different BM locations. **a** Proliferation of hMSCs calculated as PD/day from P1 to P2, donor and location dependent. **b** Proliferation average for all the donors. **c** CFU potential of hMSCs, donor and location dependent. **d** CFU average for all the donors. **e** ECM production, quantification of nodule size area in mm^2^ after cell condensation, donor and location dependent. **f** ECM production average. **g** Osteogenic potential calculated as percentage of ALP positive colonies within the CFUs, donor and location dependent. **h** Osteogenesis average. **i** Adipogenic potential, quantification of Oil red O staining relative to 100% Oil red O staining solution, donor and location dependent. **j** Adipogenesis average. The uniform distribution of data, to test inter-donor variation, was assessed using Chi-squared test and presented as a line above all donors. Values are represented as mean ± standard deviation of at least three independent experiments (n ≥ 3). Statistically significant differences were found with ***p < 0.001, **p < 0.01 and *p < 0.05

Proliferation, self-renewal ECM production and multilineage potential (osteo- and adipogenic) were assessed for hMSCs isolated from the different locations (Fig. 3). Proliferation capacity and ECM production of hMSC was similar between the different donors regardless of the BM aspiration location (Fig. 3a, e). An average for all donors showed a statistical significant increase in proliferation of hMSCs isolated from distal femur (0.64 ± 0.07) and proximal tibia (0.71 ± 0.08) when compared to the ilium (0.47 ± 0.09) and proximal femur (0.48 ± 0.13) (Fig. 3b), however no statistical significant differences were seen in ECM production (Fig. 3f).

In contrast, the CFU capacity of hMSCs showed a non-uniform distribution for all the donors, independent of the BM location (Fig. 3c). An average for all the donors showed a trend towards a higher CFU capacity of hMSCs isolated from the proximal femur 54 ± 42 CFU than ilium 31 ± 22 CFU, distal femur 14 ± 24 CFU and proximal tibia 19 ± 5 CFU (Fig. 3d). The obtained p values can be visualized in Additional file 1: Table S2.

Similarly, the mineralization capacity showed a similar trend with a higher CFU-Ob potential in hMSCs isolated from the ilium 12 ± 11 CFU-Ob (36% ± 18 CFU-Ob/CFU) and proximal femur 11 ± 10 CFU-Ob (26% ± 16 CFU-Ob/CFU) than distal femur 1 ± 1 CFU-Ob (13% ± 20 CFU-Ob/CFU) and proximal tibia 5 ± 11 CFU-Ob (17% ±22 CFU-Ob/CFU) (Fig. 3h). The obtained p values can be visualized in Additional file 1: Table S2. The high standard deviation is attributed to the non-uniform distribution over the donors (Fig. 3g).

The adipogenic potential of hMSCs showed a uniform distribution for all the donors for BM aspirated from the ilium and proximal femur but not from the distal femur and proximal tibia (Fig. 3i). An average for all the donors showed a significant increase in fat droplets in the proximal tibia 21% ±6.85 when compared to the ilium 9% ±2.5. No statistical significant differences were observed between the other groups (Fig. 3j).

### Effect of acoustic stimulation on hMSCs

Self-renewal, proliferation, ECM production and multilineage potential (osteo- and adipogenic) were assessed from the acoustic stimulated hMSCs.

**Fig. 4:**
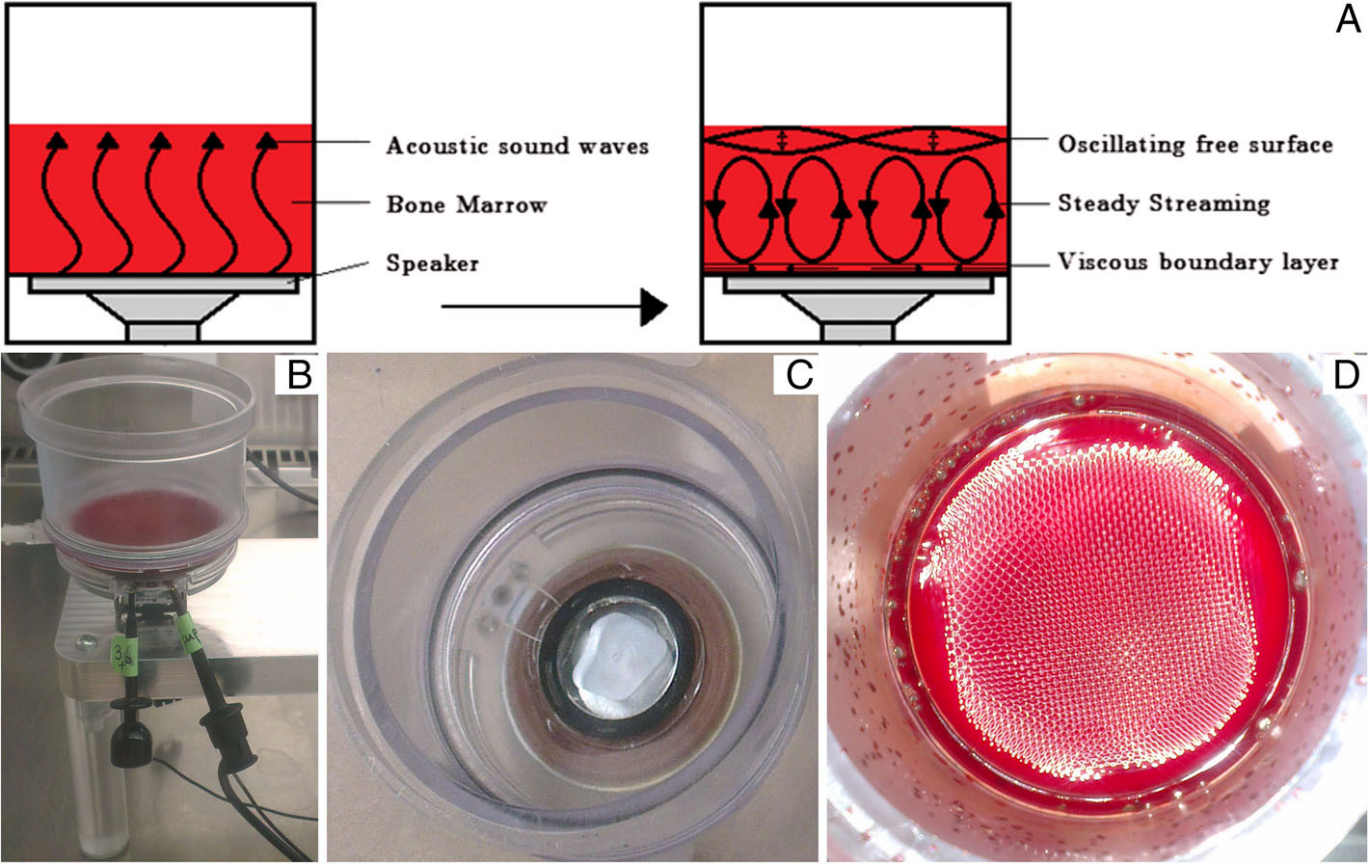
Acoustic stimulating device. **a** Sketch of the fluid flow within the processing chamber and the formation of a standing wave. **b** Processing chamber. **c** Speaker, in white, located on the bottom of the processing chamber. **d** Standing wave pattern formed in bone marrow at 300Hz

Different BM volumes harvested from different donors - 11.5 (donor 3), 10 (donor 5), 8 (donor 4), 6 (donor 1), and 5 ml (donor 2) (Additional file 3: Figure S2A) - were stimulated at a frequency of 300 Hz for 5 and 10 min (Additional file 3: Figure S2B-F). Upon acoustic stimulation a significant increase in CFU, mineralization and adipogenesis was observed for hMSC isolated from small BM volumes (5 and 6 mL) compared to larger volumes (8, 10 or 11.5 ml). No statistical significant differences were observed in proliferation or ECM production between the conditions. Based on the above-mentioned results subsequent experiments were performed using small BM volumes (4 ml). An illustration of the device, while 4 ml of BM is acoustic stimulated at 300Hz, is presented in Fig. 4.

**Fig. 5:**
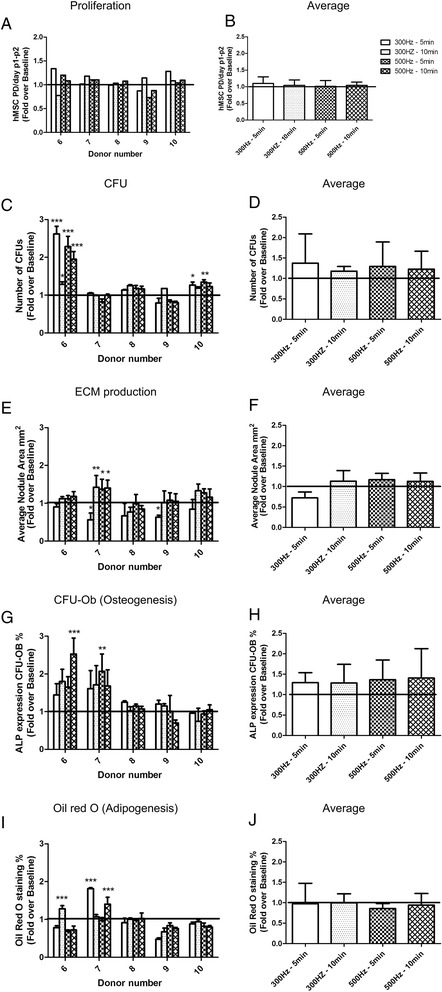
Biological characterization of isolated hMSCs from acoustic stimulated BM at 300 and 500Hz for 5 and 10 min. The results are presented as the fold change over the non-stimulated BM (baseline). **a** Proliferation of hMSCs calculated as PD/day from P1 to P2, donor and stimulation dependent. **b** Proliferation average. **c** CFU potential of hMSCs, donor and stimulation dependent. **d** CFU average. **e** ECM production, quantification of nodule size area in mm^2^, donor and stimulation dependent. **f** ECM production average. **g** Osteogenic potential calculated as percentage of ALP positive colonies within the CFUs, donor and stimulation dependent. **h** Osteogenesis average. **i** Adipogenic potential, quantification of Oil red O staining relative to 100% Oil red O staining solution, donor and stimulation dependent. **j** Adipogenesis average. Values are represented as mean ± standard deviation of at least three independent experiments (n ≥ 3). Statistically significant differences were found with ***p < 0.001, **p < 0.01 and *p < 0.05

Acoustic stimulation of BM at 300 and 500 Hz for 5 and 10 min did not change  hMSC proliferation between the conditions (Fig. 5A, B). In contrast, an increase (not significant) in CFU, ECM production and mineralization but not in adipogenic potential was observed upon acoustic stimulation (Fig. 5c -j).

Surface marker expression on hMSCs isolated from acoustic stimulated BM (300Hz for 5 min) showed a decrease, however not statistically significant, in expression of positive surface markers such as CD105 (22 ± 3% versus 32 ± 17%), CD90 (21 ± 5% versus 23 ± 7%), CD146 (3 ± 1% versus 4 ± 1%) and CD73 (20 ± 8% versus 23 ± 17%), when compared to the baseline (Additional file 4: Figure S3).

### Effect of varying the initial culture condition on hMSCs

**Fig. 6:**
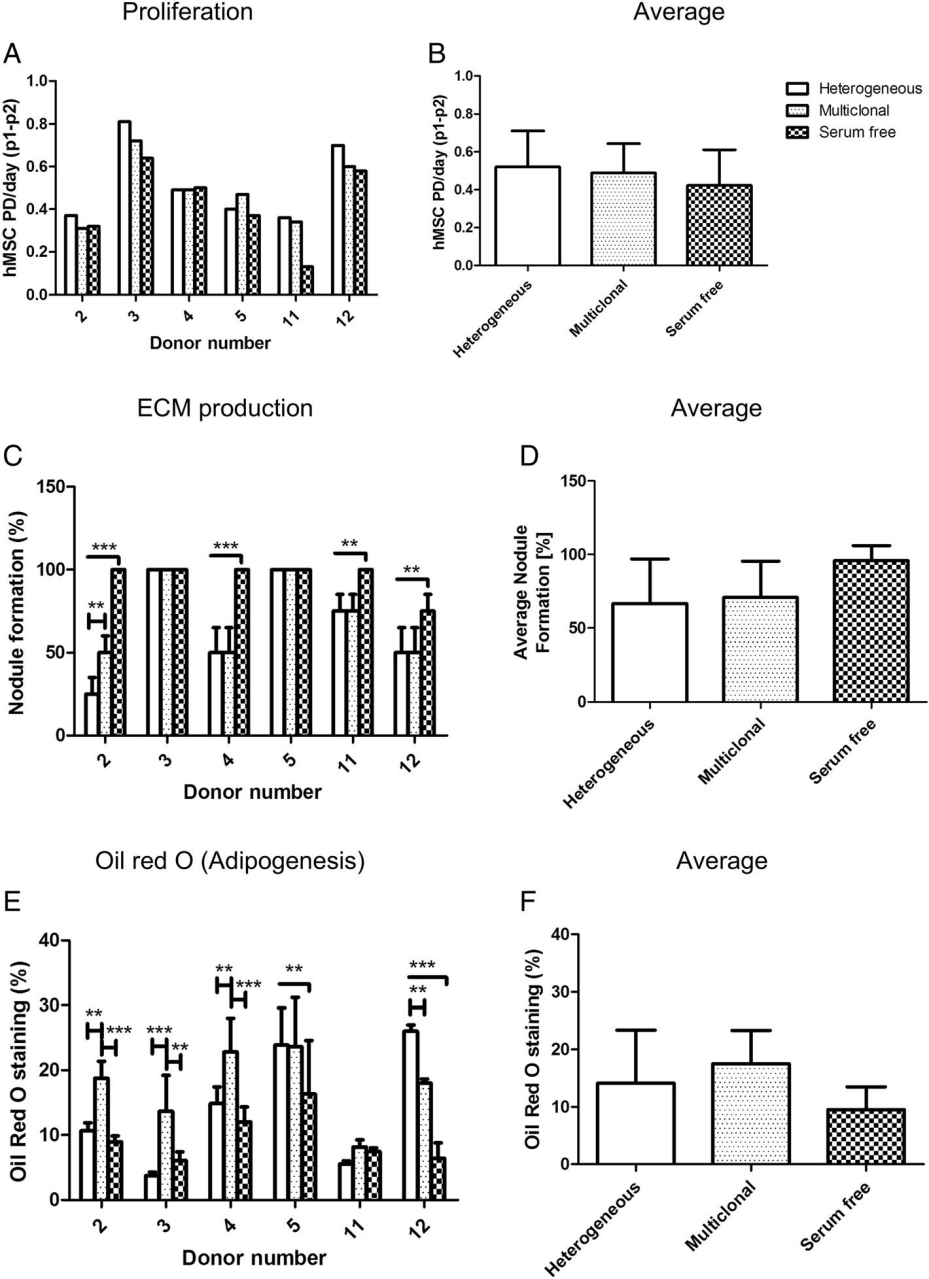
Biological characterization of hMSCs isolated from BM under different isolation procedures **a** Proliferation of hMSCs calculated as PD/day from P1 to P2, donor and isolation procedure dependent. **b** Proliferation average. **c** ECM production, percentage of formed nodules, donor and isolation procedure dependent. **d** ECM production average. **e** Adipogenic potential, quantification of Oil red O staining relative to 100% Oil red O staining solution, donor and isolation procedure dependent. **f** Adipogenesis average. Values are represented as mean ± standard deviation of at least three independent experiments (n ≥ 3). Statistically significant differences were found with ***p < 0.001 and **p < 0.01

The isolation of hMSCs from the BM was assessed by varying the initial culture conditions and their proliferation, ECM production, multilineage differentiation potential and cell surface marker expression was analyzed. No difference in proliferation (Fig. 6a, b and Additional file 1: Table S3) and osteogenesis (Additional file 5: Figure S4) was observed between the different isolation conditions. In contrast, isolation of hMSCs under SF condition showed a trend in increased ECM production, with 4 out of 6 donors showing statistically significant increase (Fig. 6c). When averaged for  all the donors, p values of 0.11 and 0.18 were obtained when compared to heterogeneous and multiclonal conditions (Fig. 6d and Additional file 1: Table S3). Additionally, SF condition showed a trend in decreased adipogenesis, with 5 out of 6 donors showing a statistically significant decrease (Fig. 6e). When averaged for all the donors p values of 0.48 and 0.13 were obtained when compared to heterogeneous and multiclonal conditions (Fig. 6f and Additional file 1: Table S3). Isolation of hMSCs under multiclonal condition showed a trend towards increased adipogenesis (Fig. 6e). Statistically significant increase in adipogenesis was observed in 5 out of 6 donors in multiclonal when compared to SF isolated hMSCs (Fig. 6e and f, Additional file 1: Table S3).

The expression of CD271, CD34, CD14, CD79a, CD45 and HLA-DR was absent in all conditions regardless of the isolation procedure, while no significant differences where observed in the expression of CD90 (46 ± 31% heterogeneous versus 36 ± 26% multiclonal and 43 ± 36% serum free condition), CD105 (11 ± 6% heterogeneous versus 23 ± 14% multiclonal and 34 ± 31% serum free condition), CD73 (27 ± 7% heterogeneous versus 33 ± 18% multiclonal and 43 ± 33% serum free condition) and CD146 (5 ± 4% heterogeneous versus 5 ± 4% multiclonal and 11 ± 16% serum free condition) between the isolation conditions. However, a trend towards higher expression of CD105 (p =0.28), CD73 (p = 0.57) and CD146 (p = 0.66) was observed in the hMSCs isolated in SF media when compared to the heterogeneous condition (Additional file 6: Figure S5). The high standard deviation is the result of inter-donor variation.

## Discussion

The human body has an extensive capacity to regenerate bone tissue after trauma. However, large defects cannot be restored without intervention and often lead to nonunion. Long bone fracture repair has been extensively studied at both clinical as well as fundamental level, however little is known about the differences in fracture repair between the femur and the tibia [6, 7]. Therefore the aim of the present study was to assess the pool and biological functions of BM-derived hMSCs in the lower limbs, such as the ilium, proximal femur, distal femur and proximal tibia. Additionally, we broadened our research interest towards methods to prime BM-derived hMSCs for later reimplantation at the fracture site. This should facilitate their homing and commitment towards a faster bone regeneration, as it has been already shown that a reduced pool of proliferative and multipotent hMSCs are present at the low healing fractures [23].

In the present study we showed that the pool of BM-derived hMSCs differ with respect to the BM aspiration location. We found that after 14 days the number of hMSCs isolated from the ilium and proximal femur was higher (Fig. 2b) and they showed higher self-renewal and osteogenic differentiation potential (Fig. 3d, f) in comparison to hMSCs isolated from the distal femur and proximal tibia, with the latter showing higher adipogenic potential. These findings correspond to the macroscopic appearance of the BM as described by Malkiewicz et al. [24], with red BM found in the ilium and proximal femur, suggesting an active participation to hematopoiesis, and yellow BM found in distal femur and proximal tibia, which is enriched in adipocytes. During aging, red marrow is replaced by yellow marrow and this change in the marrow compartment might contribute to differences in the fracture repair cascade [24]. In this context, we strongly believe that the differences in BFH rate between femur and tibia are the result of insufficient amount of hMSCs present at the fractured site, as well as their poor self-renewal and osteogenic potential. Additionally, previous studies demonstrated the use of bone marrow aspirate and its efficacy in the treatment of fracture nonunion or high nonunion rate repair [25, 26]. Therefore, we propose that the isolation of BM from the ilium, and its delivery in tibial fractures in order to enhance bone healing, could improve the current clinical treatment strategy.

In the process of quantifying the concentration of MNCs with regard to the aspirated BM volume, we found that 10 ml of BM yields the highest MNC concentration. Higher BM volumes yielded low concentrations of MNCs, due to the dilution with peripheral blood during aspiration, while lower BM volumes yielded also lower concentration of MNCs, as described by Fennema EM et al. [27]. Interestingly, in both studies the same average concentration of MNCs (2.6*10^7^ MNCs/ml) was found for 10 mL aspirates, henceforth encouraging the surgeons to limit the aspirated BM volume from the ilium to 10 ml.

In order to increase the contribution of cells to bone repair, a new paradigm emerged in tissue regeneration, focusing on rhythms and oscillatory patterns capable of orchestrating cell fate decision. The use of physical energy, such as ultrasound vibration has shown to affect the cell fate and increase the rate of bone repair [7, 28, 29], however the therapy has been rather inefficient likely due to the low number of pro-regenerative cells present [6]. Therefore, we propose a different approach: the delivery of acoustic stimulated BM from the ilium (rich in hMSCs) at the fracture site. Based on a previous study by Ridgway J. et al., where acoustic vibration was used to separate cells from BM suspension, by trapping the cells in the pressure node planes of the standing wave and reducing the volume, an increase in CFU-Ob potential was observed in the processed BM [22]. We believe that this increase was not only due to the reduction in BM volume but also a change in cell fate. To test this we selected two different frequencies in the range of acoustic vibration, 300 and 500 Hz, and two time points 5 and 10 min. The results obtained showed a trend towards an increased self-renewal, ECM production and a shift towards osteogenic, but not adipogenic, differentiation in acoustic stimulated BM, suggesting that hMSCs may sense the acoustic vibratory frequencies. However, the long expansion period necessary to obtain sufficient cell numbers to perform the assays eventually led to a decrease in the multilineage potential, as cell potential is known to diminish with increased in vitro culture time [30, 31]. In addition, we speculate that the decrease in positive hMSCs surface markers in acoustically stimulated BM is the result of integrin reorganization (cellular mechanoreceptor on the cell surface), followed by surface markers reorganization [32] and change in cell fate. To our knowledge this is the first study where acoustic energy was applied directly to BM and not on cultured cells paving the way to its implementation into a one-step surgical procedure for bone repair. The harvested BM can be first exposed to acoustic stimulation during the surgical intervention followed by administration to the fracture zone in cases where the risk of nonunion is high or in revision surgeries for pseudarthrosis.

While acoustic sound vibration focuses on changing the phenotype of the cells, variation of the initial hMSCs isolation conditions focuses on the selection of a defined cell pool. We found that isolation of hMSCs in SF media selects a pro-ECM cell population, which could be of great help in accelerating the rebuilding process of a native ECM after a bone fracture. In contrast, we found that isolation of hMSCs using low MNC plating densities selects a pro-adipogenic cell population. These findings underline the importance of carefully selecting the right isolation procedure for the right application.

## Conclusion

Overall, our results suggest that novel approaches to bone fracture healing can be developed based on our improved understanding of bone marrow cell biology. Based on our results we hypothesize that poor BFH in the tibia might be the result of insufficient cell numbers as well as their poor osteogenic potential. Based on this we suggest the aspiration of BM from the ilium and its delivery into the tibia to accelerate fracture healing. Moreover, we proposed two new possible therapeutic approaches for BFH: acoustic stimulation of BM and use of preselected pro-ECM hMSCs pool for delivery at the fracture site.

## Additional files

Additional file 1: Table S1. Testing the significance in the number of hMSCs isolated from different BM aspiration location. The identified p value is presented after performing one way ANOVA and Tukey’s multiple comparison test. **Table S2.** Testing the significance of difference in CFUs and CFU-Ob from different BM aspiration location. The identified p value is presented after performing one way ANOVA and Tukey’s multiple comparison test. **Table S3.** Testing the significance in proliferation, ECM production and adipogenesis of different hMSCs isolation and cell culture conditions. The identified *p* value is presented after performing one way ANOVA and Tukey’s multiple comparison test. (PDF 85 kb)
Additional file 2: Figure S1. Macroscopic appearance of bone marrow aspirated from different locations: ilium, proximal femur, distal femur and proximal tibia. (PDF 311 kb)
Additional file 3: Figure S2. Biological characterization of isolated hMSCs from acoustically stimulated BM at 300 Hz for 5 min at different volumes, 11.5, 10, 8, 6 and 5 ml. The results are presented as the fold change over the non-stimulated bone marrow (baseline). (A) Graphic representation of the bone marrow volumes, donor dependent. (B) Proliferation of hMSCs calculated as PD/day from P1 to P2, donor and volume dependent. (C) CFU potential of hMSCs, donor and volume dependent. (D) ECM production, quantification of nodule size area in mm^2^, donor and volume dependent. (E) Osteogenic potential calculated as percentage of ALP positive colonies within the CFUs, donor and volume dependent. (F) Adipogenic potential, quantification of Oil red O staining relative to 100% Oil red O staining solution, donor and volume dependent. Values are represented as mean ± standard deviation of at least three independent experiments (n ≥ 3). Statistically significant differences were found with ***p < 0.001, **p < 0.01 and **p* < 0.05. (PDF 694 kb)
Additional file 4: Figure S3. Surface marker expression (in percentage) of the acoustic stimulated cells represented as a bar plot. Each bar represents the average expression obtained from three independent donors. Represented are only the surface markers that were expressed in the obtained populations. Negative markers are not shown. No statistically significant differences were found between the two conditions. (PDF 184 kb)
Additional file 5: Figure S4. Alizarin red staining of calcium nodules after osteogenic induction of hMSC isolated under varying culture condition from different donors. No differences were observed between the culture conditions, though differences between the donors were identified. Donor 2 and 11 showed less calcium nodules formation than the rest of the donors. All the controls stained negative for calcium nodules formation. Values are represented as mean ± standard deviation of at least three independent experiments (*n* = 3). (PDF 2096 kb)
Additional file 6: Figure S5. Surface marker expression (in percentage) of the varying culture conditions represented as a bar plot. Each bar stands for the average over the percentage of surface markers obtained from three donors. Selected sets of cell surface markers expressed positive on hMSC. All the other investigated sets were expressed negative for both conditions, therefore not shown. Not statistically significant differences were found between the three conditions. (PDF 210 kb)

## Abbreviations

ALP: Alkaline phosphatase
BFH: Bone fracture healing
BGP: β-glycerophosphate
BM: Bone marrow
CFU: Colony forming unit
CFU-Ob: Colony forming unit osteoblast
Dex: Dexamethasone
ECM: Extracellular matrix
FBS: Fetal bovine serum
GM: Growth media
hMSCs: Human mesenchymal stromal cells
MNCs: Mononuclear cells
P1: Passage 1
P2: Passage 2
PD: Population doubling
SF: Serum free
THA: Total hip arthroplasty
TKA: Total knee arthroplasty
α-MEM: α-minimal essential media
