# Supplementary material for: Means of enhancing bone fracture healing: optimal cell source, isolation methods and acoustic stimulation
Source: BMC Biotechnol. 2016 Dec 12;16:89. doi: 10.1186/s12896-016-0318-1 (PMC5154008; doi:10.1186/s12896-016-0318-1)
Supplement: Additional file 1: Table S1. — Testing the significance in the number of hMSCs isolated from different BM aspiration location. The identified p value is presented after performing one way ANOVA and Tukey’s multiple comparison test. Table S2. Testing the significance of difference in CFUs and CFU-Ob from different BM aspiration location. The identified p value is presented after performing one way ANOVA and Tukey’s multiple comparison test. Table S3. Testing the significance in proliferation, ECM production and adipogenesis of different hMSCs isolation and cell culture conditions. The identified p value is presented after performing one way ANOVA and Tukey’s multiple comparison test. (PDF 85 kb) [file 12896_2016_318_MOESM1_ESM.pdf]

Supplementary Table 1. Testing the significance of difference in number of hMSCs isolated from different BM aspiration location. The identified p value is presented after performing one way ANOVA and Tukey's multiple comparison test.

BM location comparison	P value
Ilium vs. proximal femur	0.36
Ilium vs. distal femur	0.07
Ilium vs. proximal tibia	0.12
Proximal femur vs. distal femur	0.86
Proximal femur vs. proximal tibia	0.94
Distal femur vs. proximal tibia	0.99

Supplementary Table 2. Testing the significance of difference in CFUs and CFU-Ob from different BM aspiration location. The identified p value is presented after performing one way ANOVA and Tukey's multiple comparison test.

Experiment	BM location comparison	P value
CFU	Ilium vs. proximal femur	0.45
	Ilium vs. distal femur	0.72
	Ilium vs. proximal tibia	0.87
	Proximal femur vs. distal femur	0.07
	Proximal femur vs. proximal tibia	0.13
	Distal femur vs. proximal tibia	0.99
CFU-Ob	Ilium vs. proximal femur	0.76
	Ilium vs. distal femur	0.1
	Ilium vs. proximal tibia	0.21
	Proximal femur vs. distal femur	0.5
	Proximal femur vs. proximal tibia	0.73
	Distal femur vs. proximal tibia	0.98

Supplementary Table 3. Testing the significance in proliferation, ECM production and adipogenesis of different hMSCs isolation and cell culture conditions. The identified p value is presented after performing one way ANOVA and Tukey's multiple comparison test.

Experiment	Cell culture condition	P value
Proliferation	Heterogenous vs. multiclonal	0.94
	Heterogenous vs. serum free	0.62
	Multiclonal vs. serum free	0.81
ECM production	Heterogenous vs. multiclonal	0.95
	Heterogenous vs. serum free	0.11
	Multiclonal vs. serum free	0.18
Oil red O (Adipogenesis)	Heterogenous vs. multiclonal	0.66

	Heterogenous vs. serum free	0.48
	Multiclonal vs. serum free	0.13
