# Supplementary figures and images for: Means of enhancing bone fracture healing: optimal cell source, isolation methods and acoustic stimulation
Source: BMC Biotechnol. 2016 Dec 12;16:89. doi: 10.1186/s12896-016-0318-1 (PMC5154008; doi:10.1186/s12896-016-0318-1)

Ilium

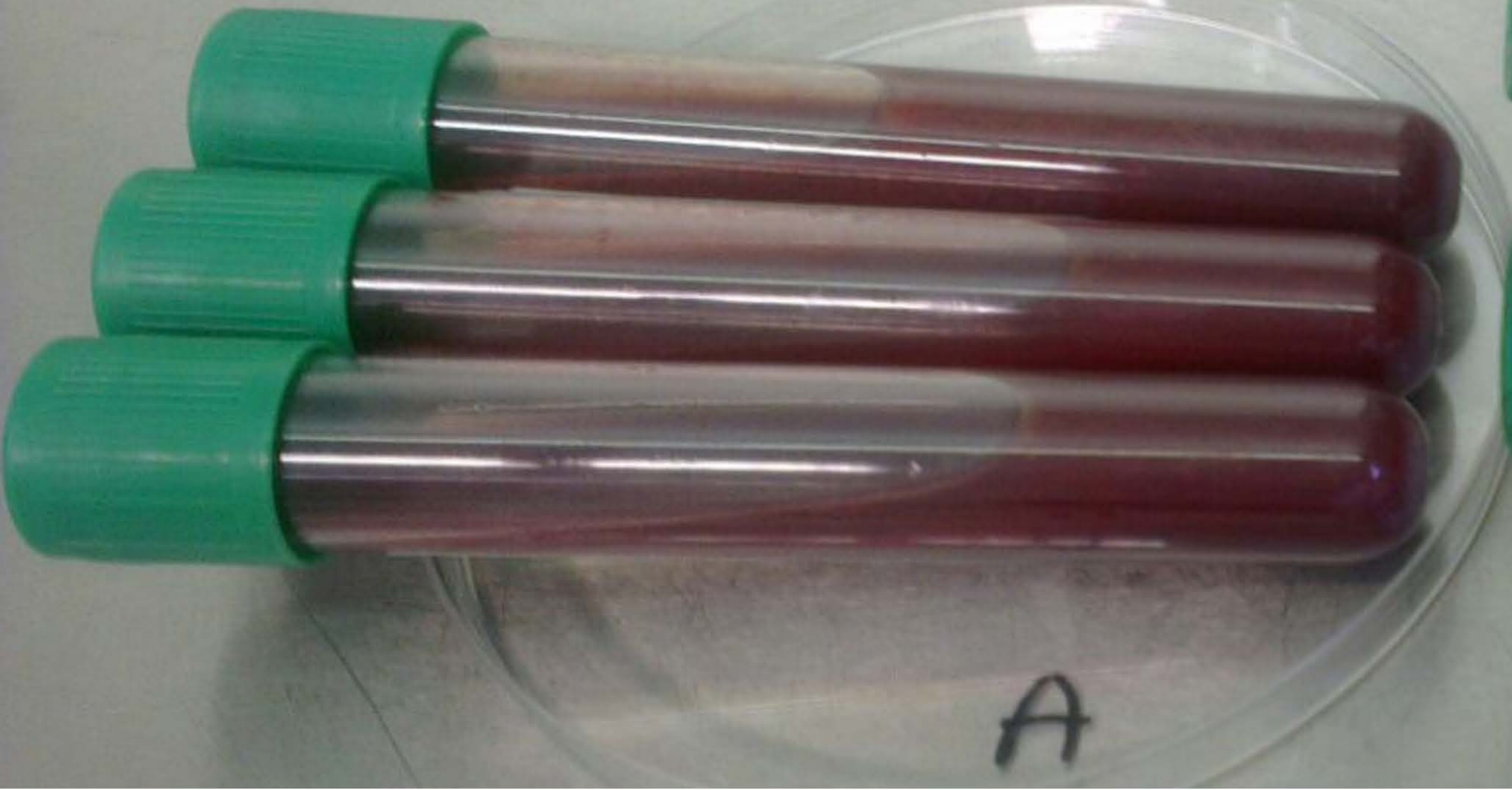

Proximal Femur

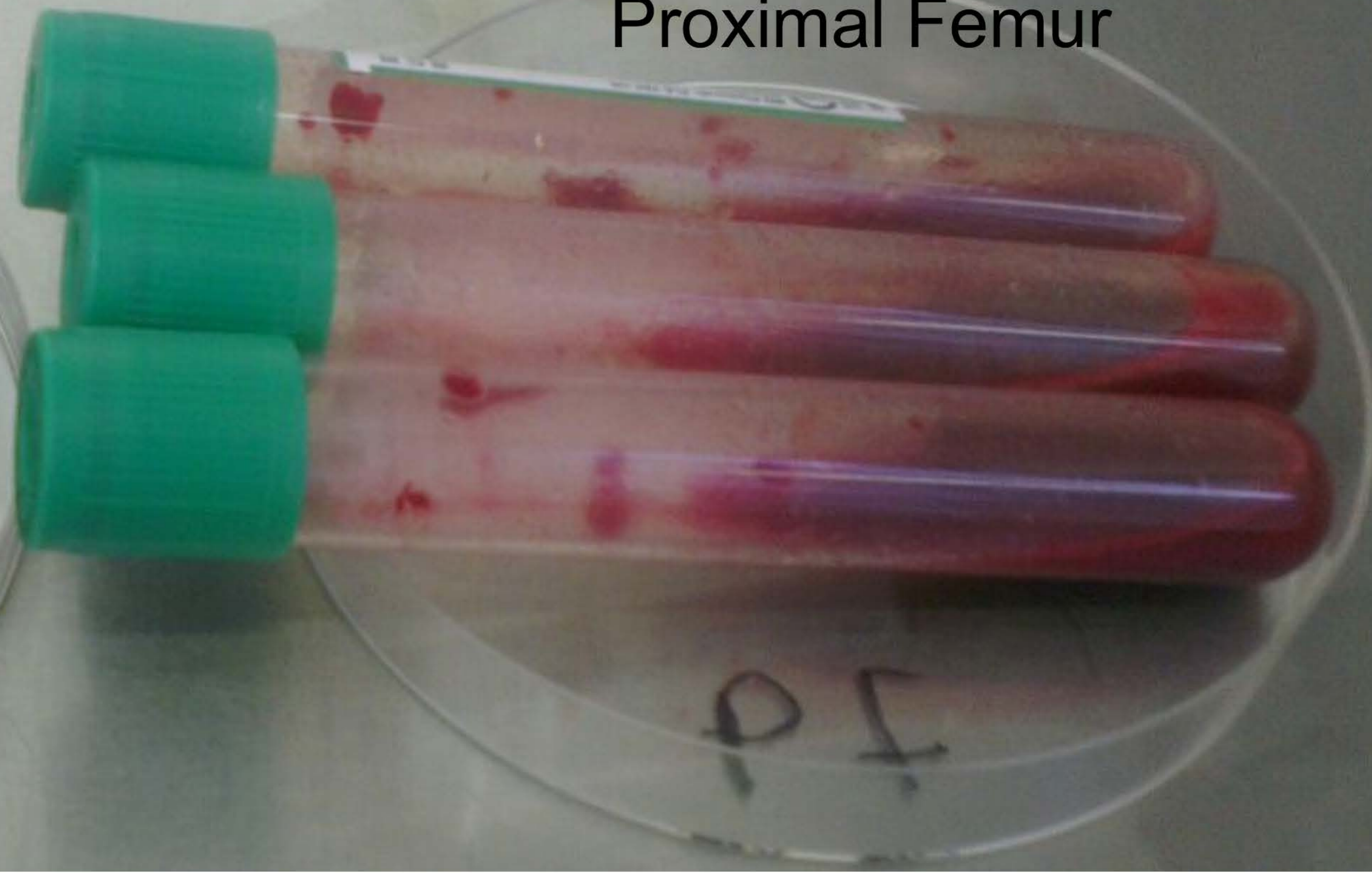

Distal Femur

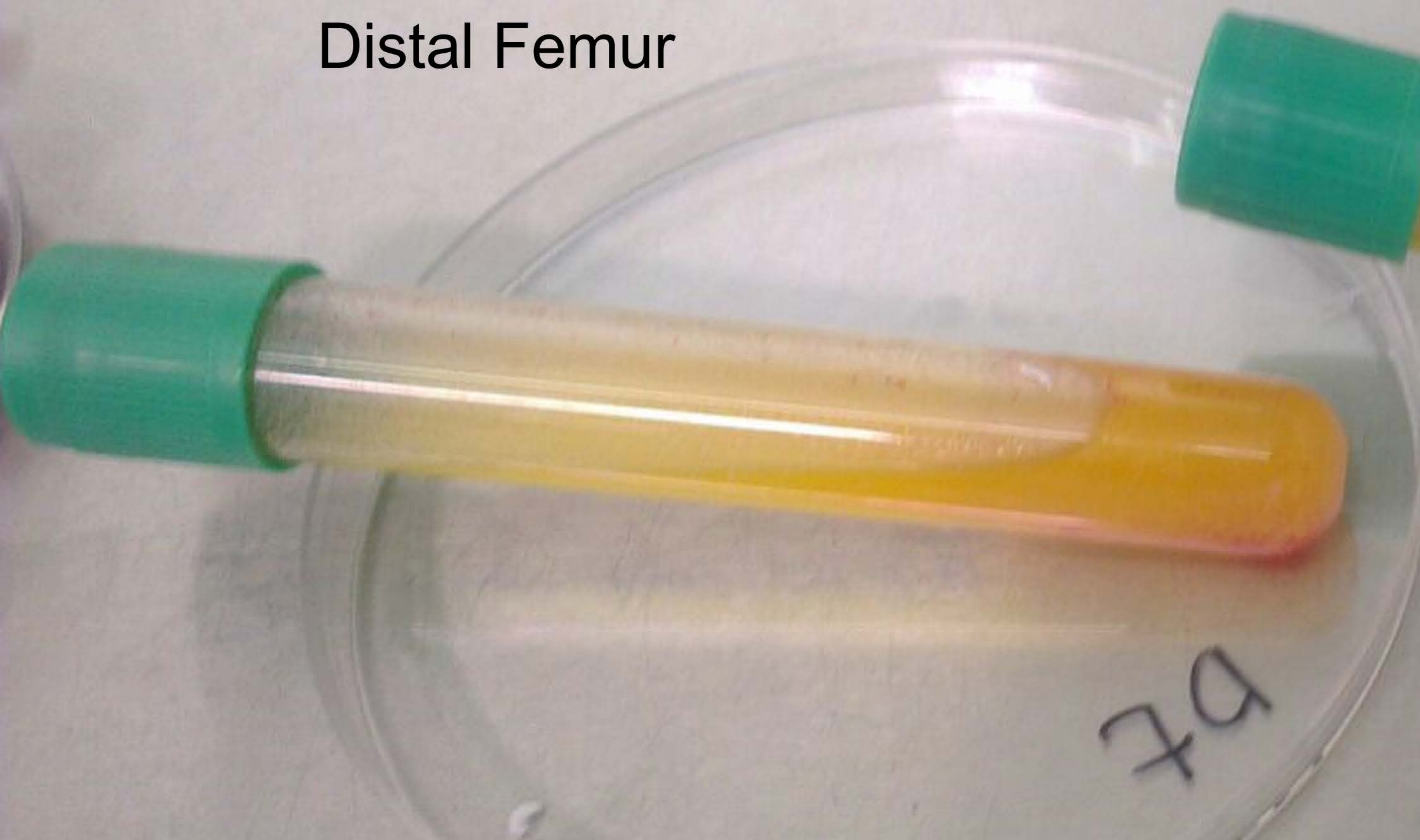

Proximal Tibia

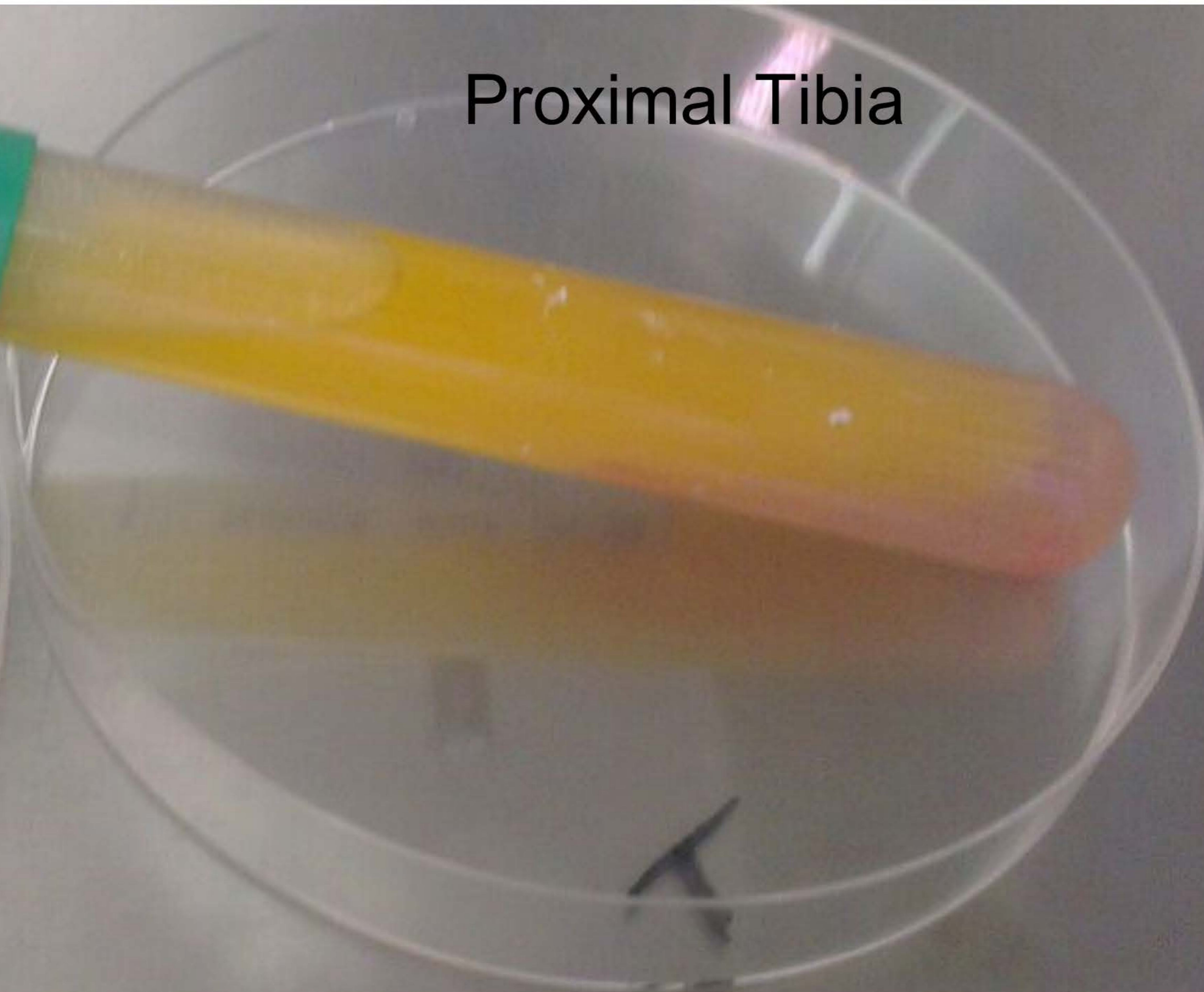

Supplement: Additional file 2: Figure S1. — Macroscopic appearance of bone marrow aspirated from different locations: ilium, proximal femur, distal femur and proximal tibia. (PDF 311 kb) [file 12896_2016_318_MOESM2_ESM.pdf]

# Surface marker expression

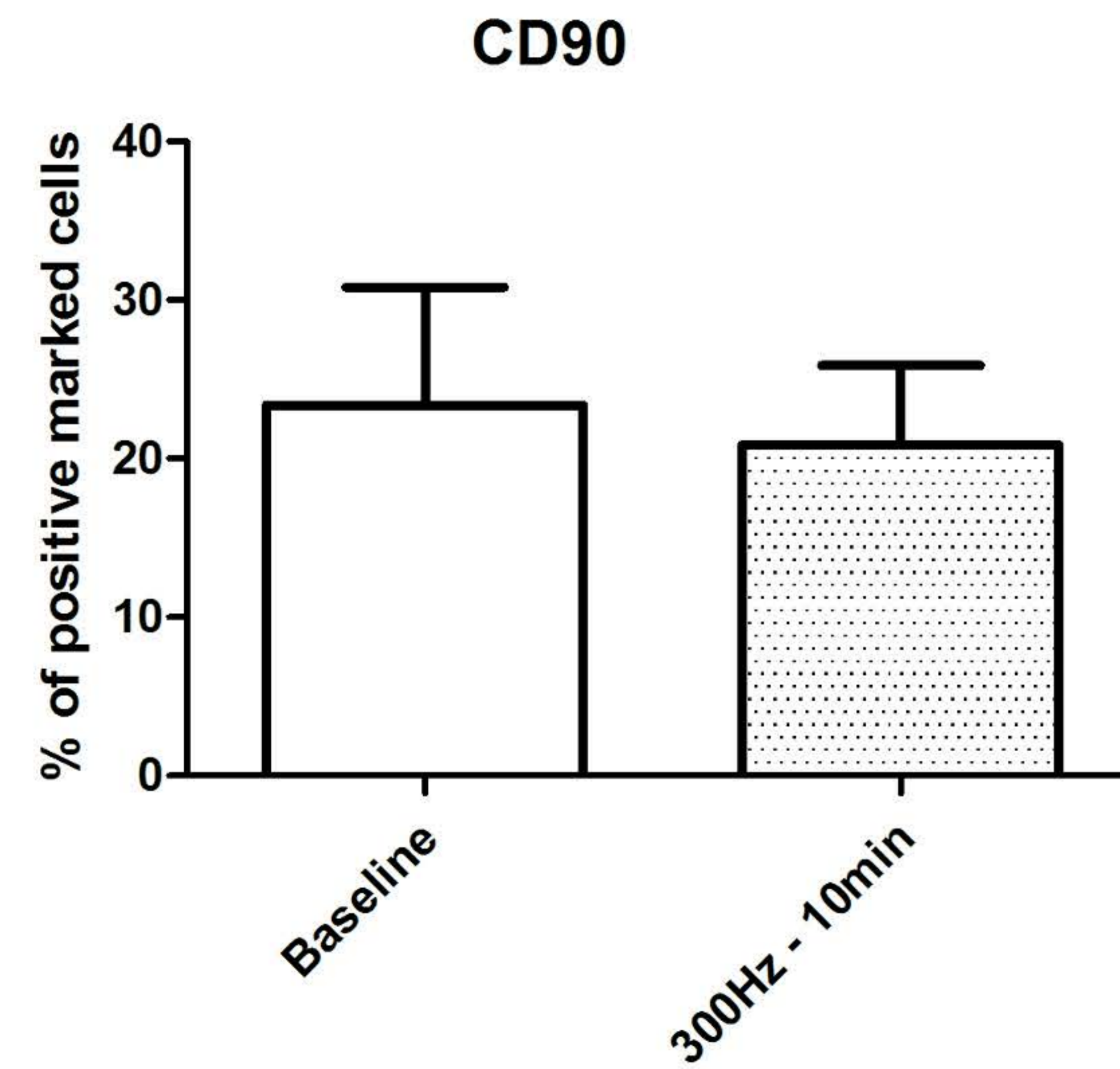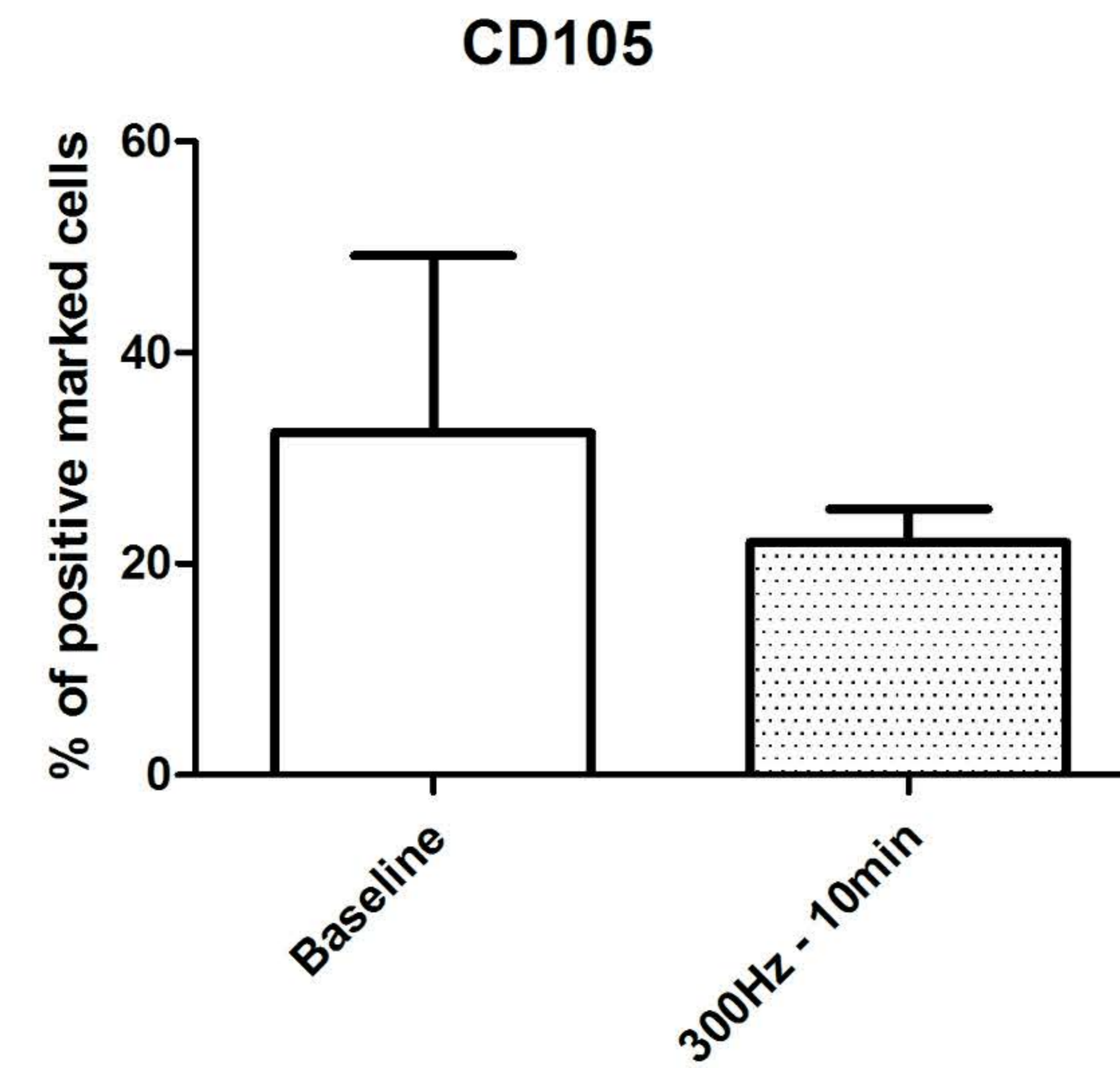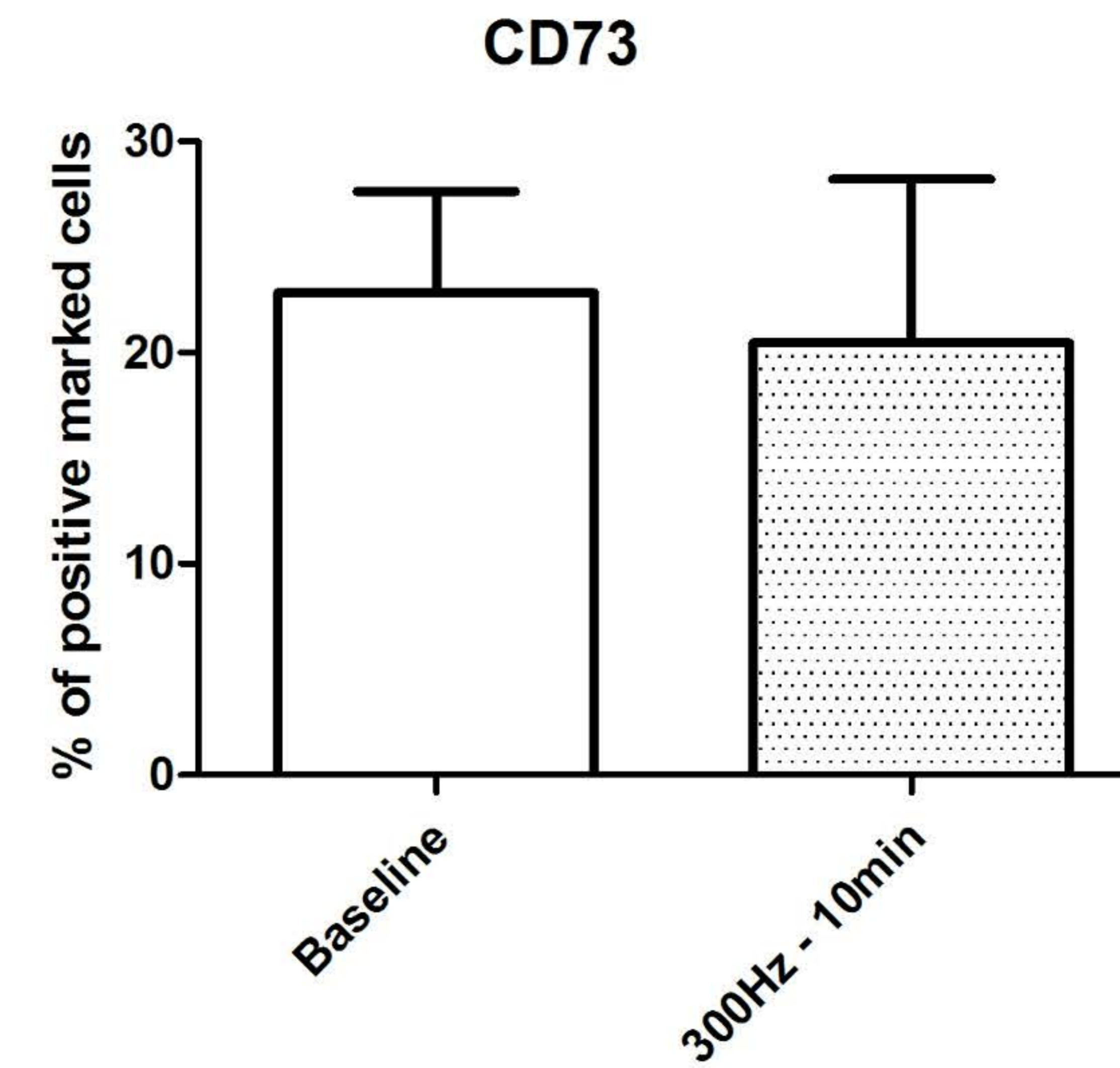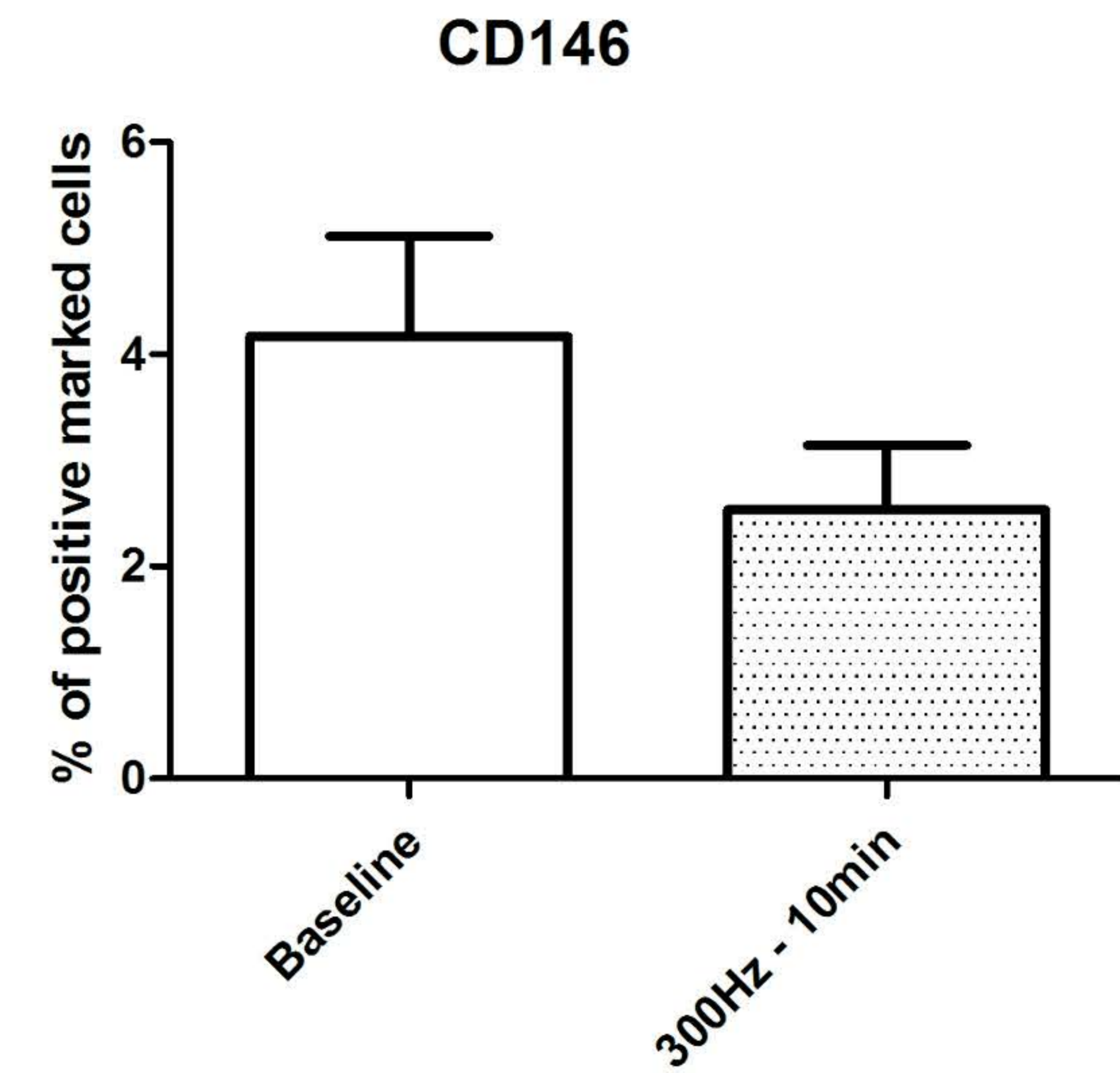

Supplement: Additional file 4: Figure S3. — Surface marker expression (in percentage) of the acoustic stimulated cells represented as a bar plot. Each bar represents the average expression obtained from three independent donors. Represented are only the surface markers that were expressed in the obtained populations. Negative markers are not shown. No statistically significant differences were found between the two conditions. (PDF 184 kb) [file 12896_2016_318_MOESM4_ESM.pdf]

# Heterogeneous

# Multilineage

SF

# Control

## Donor 2

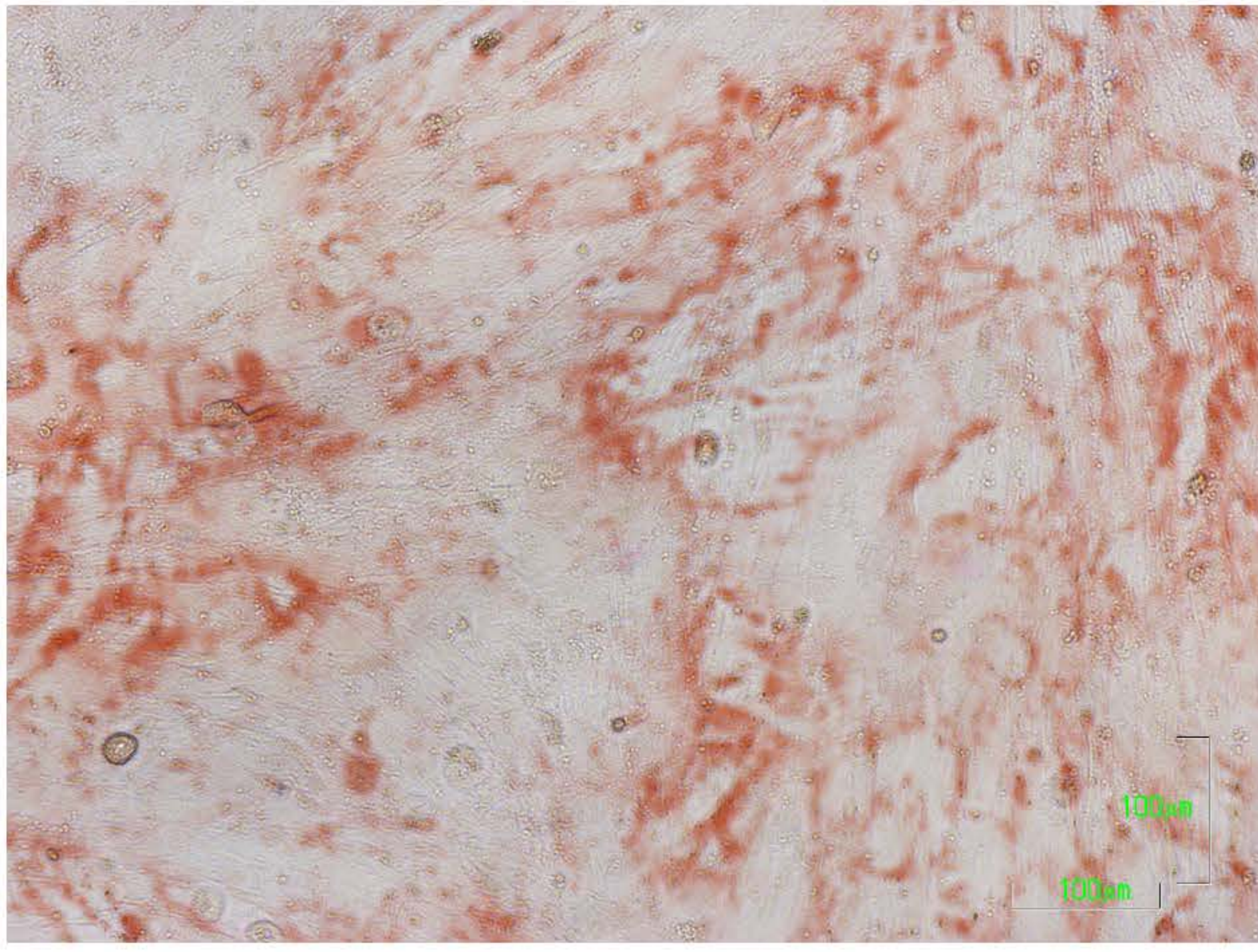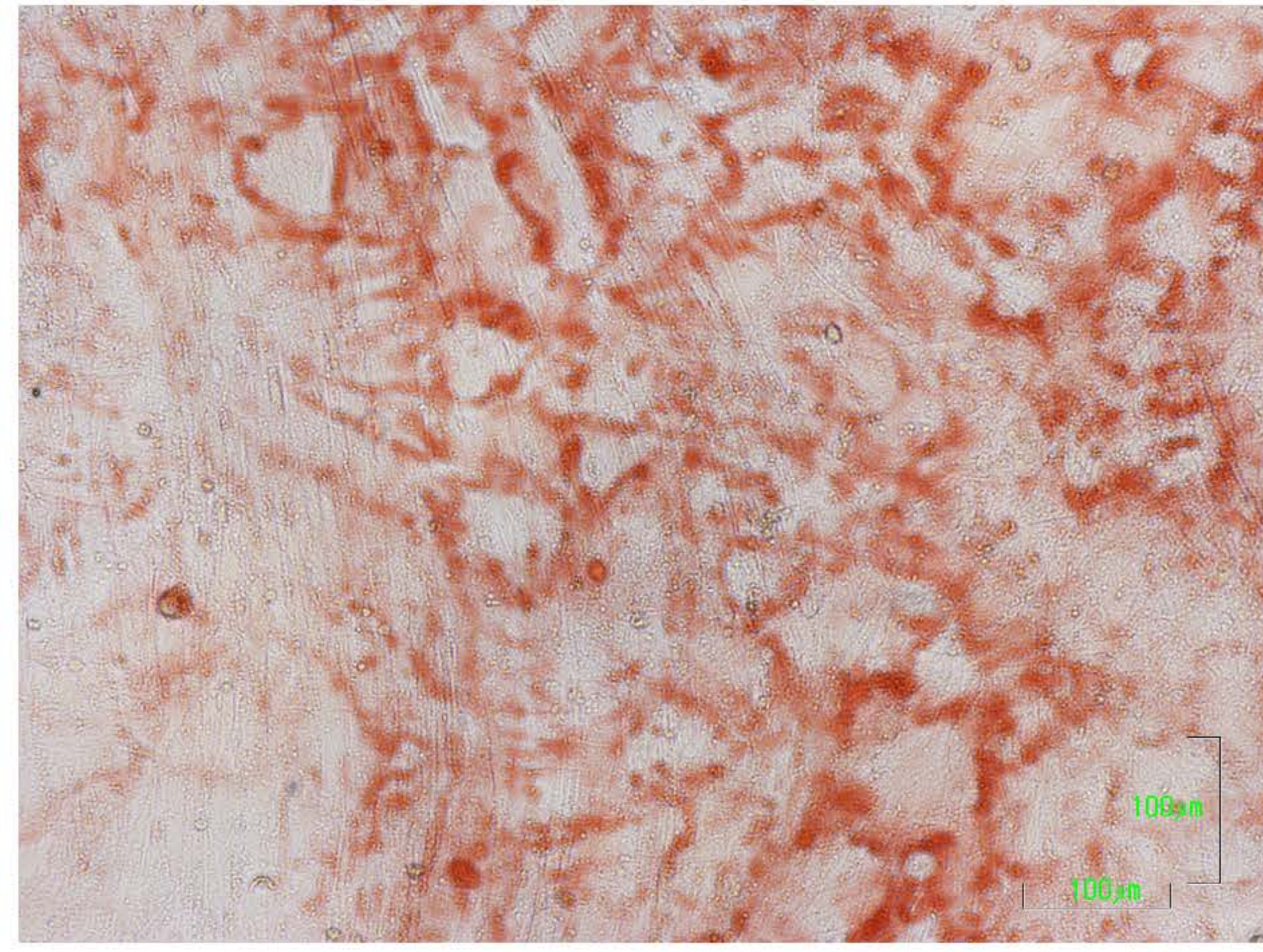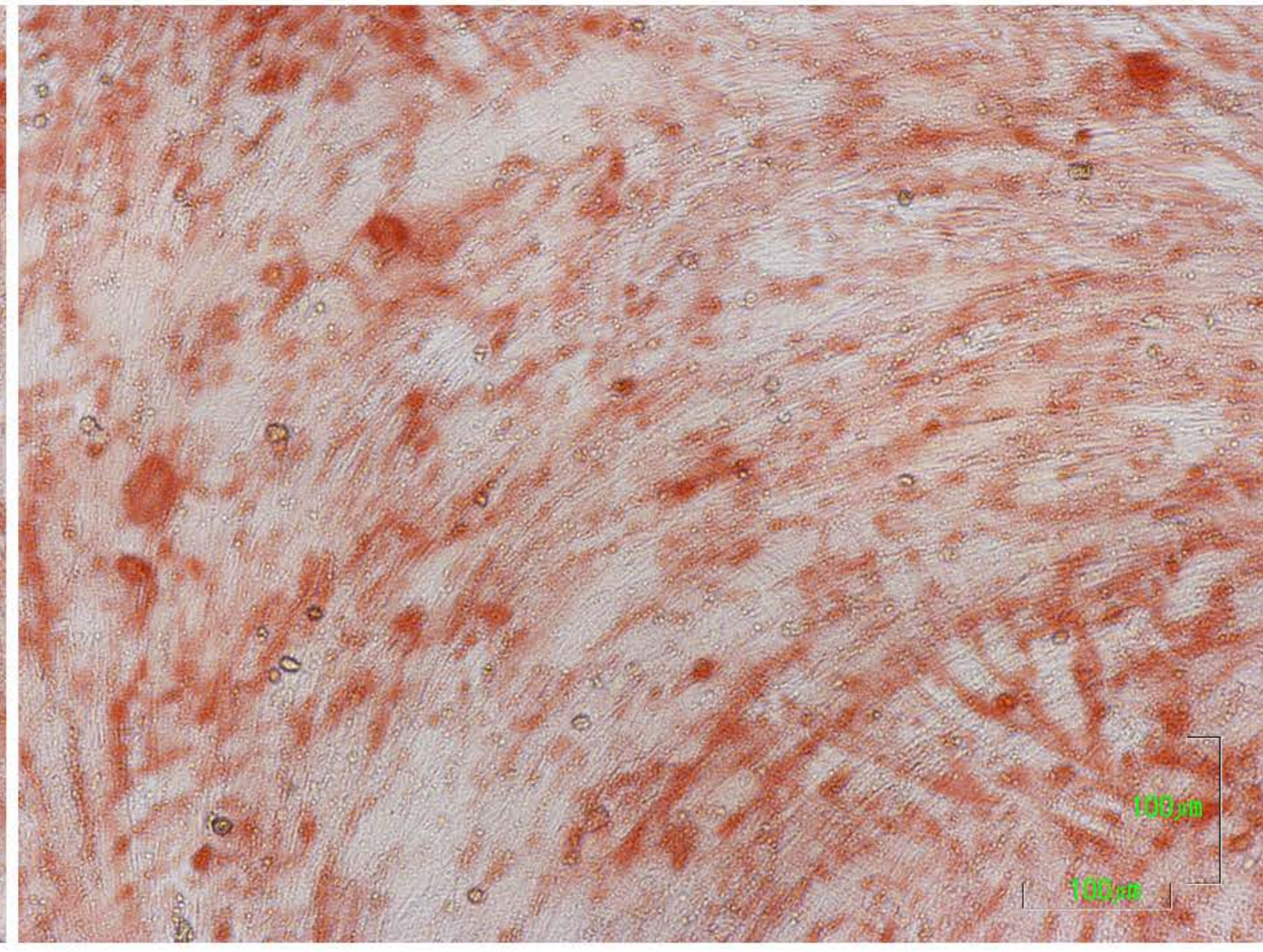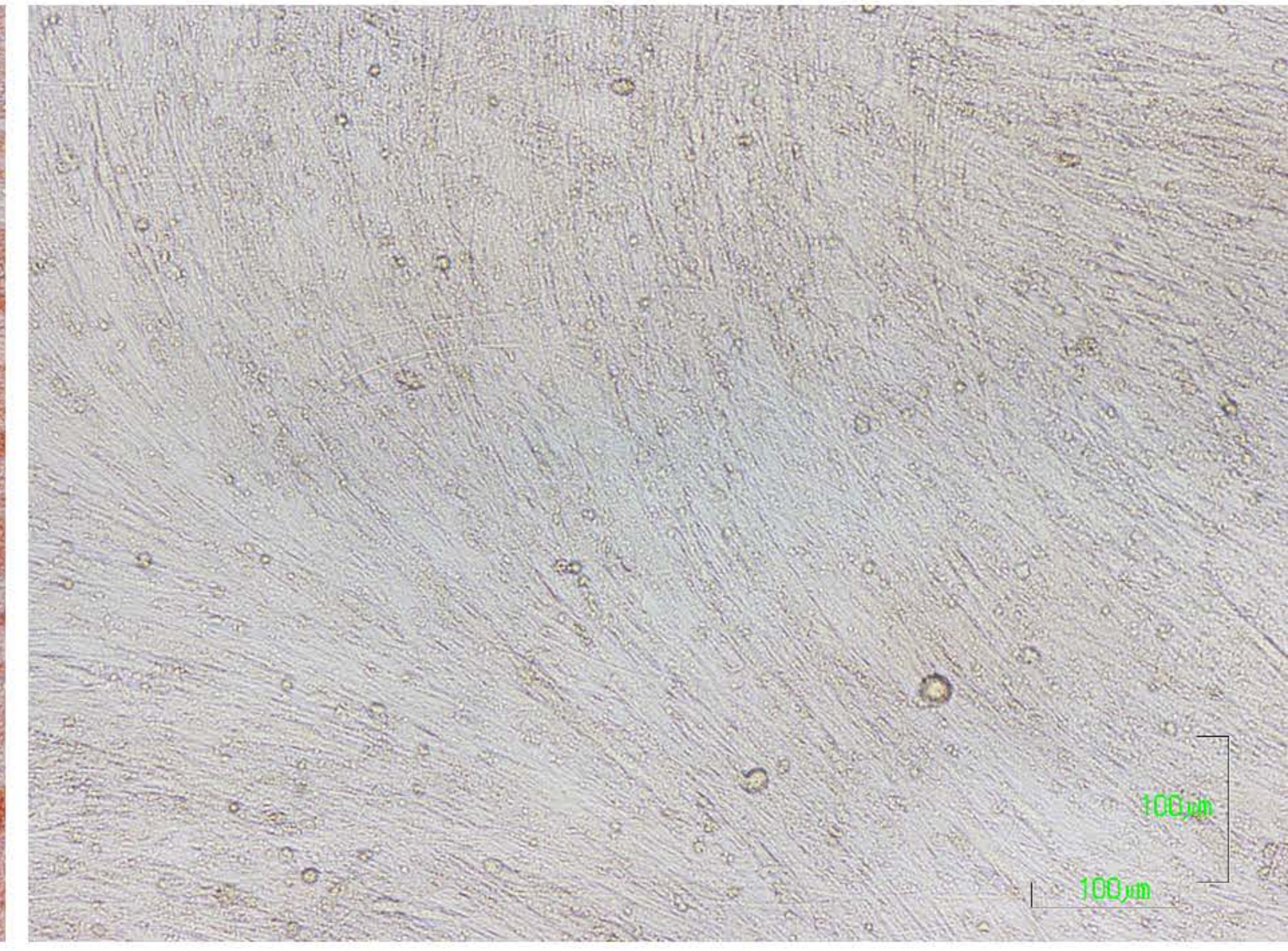

## Donor 3

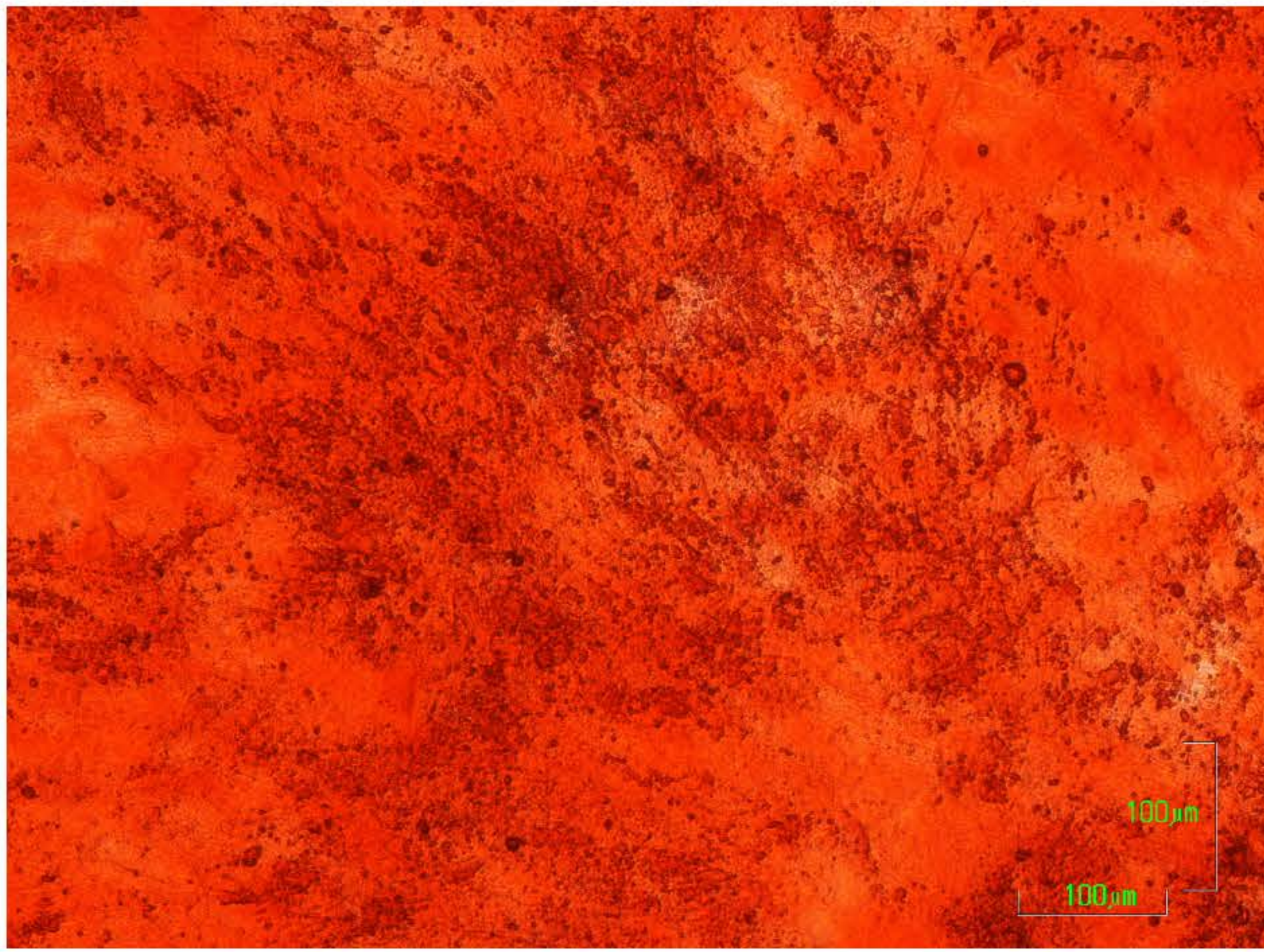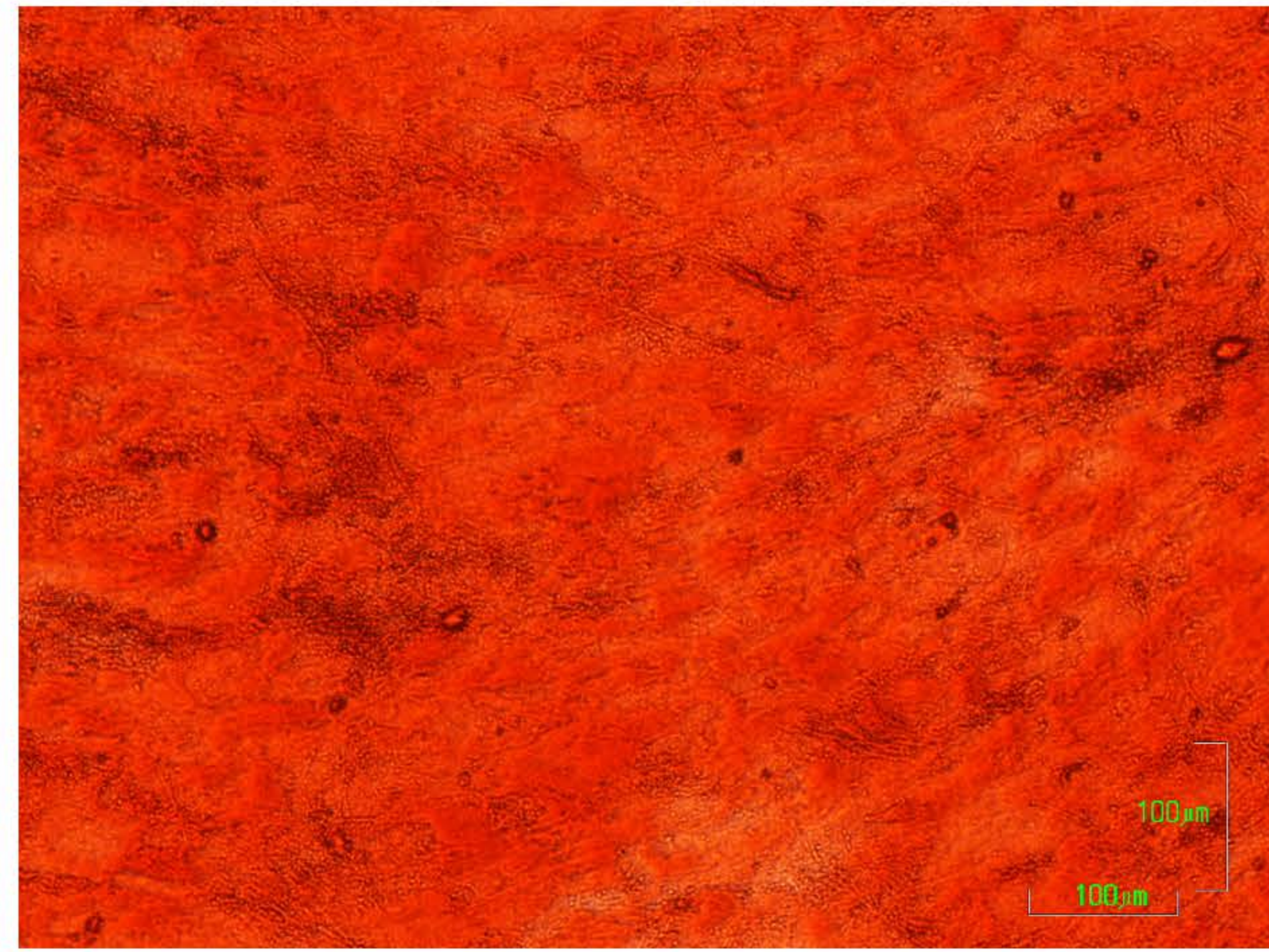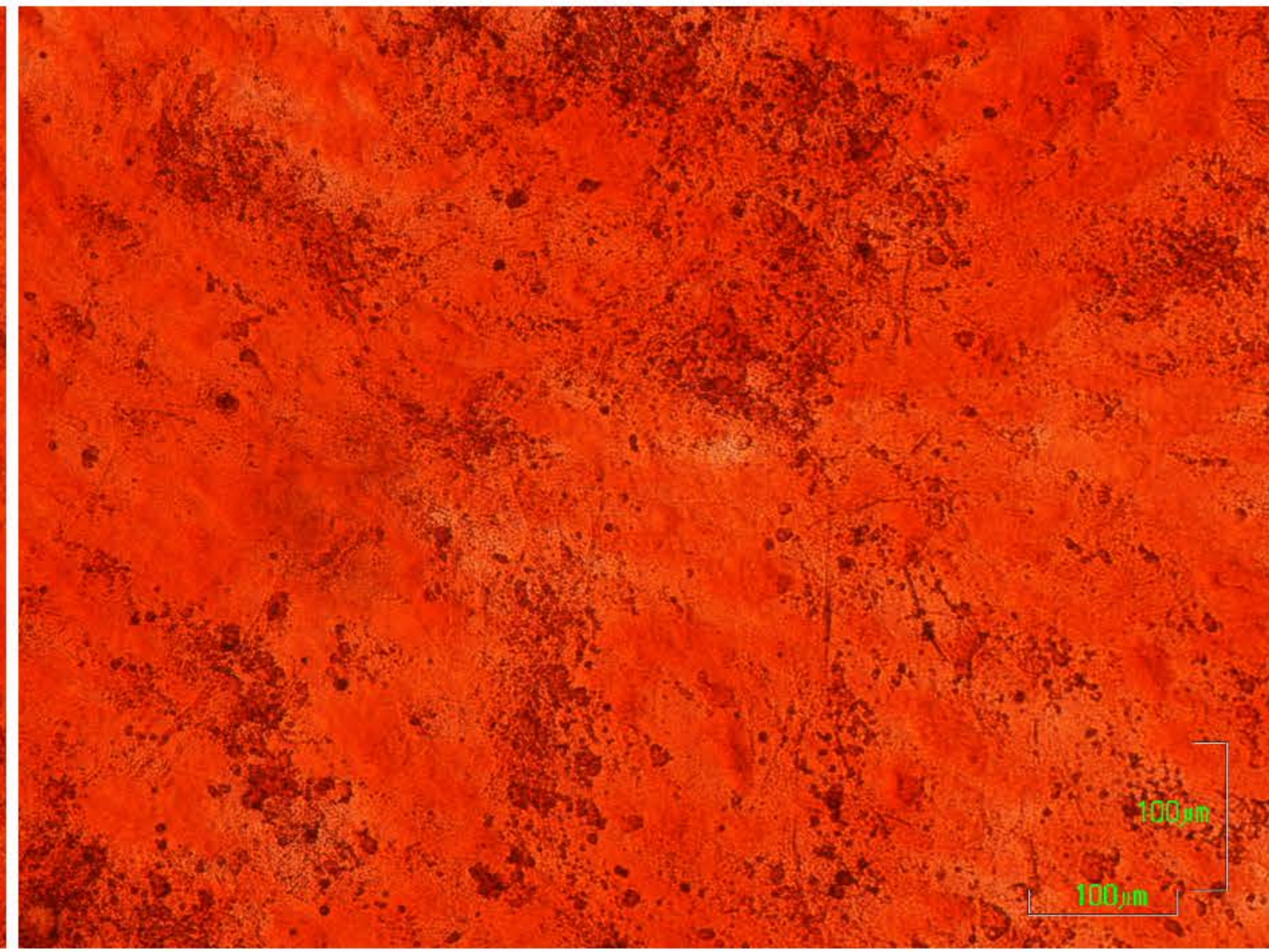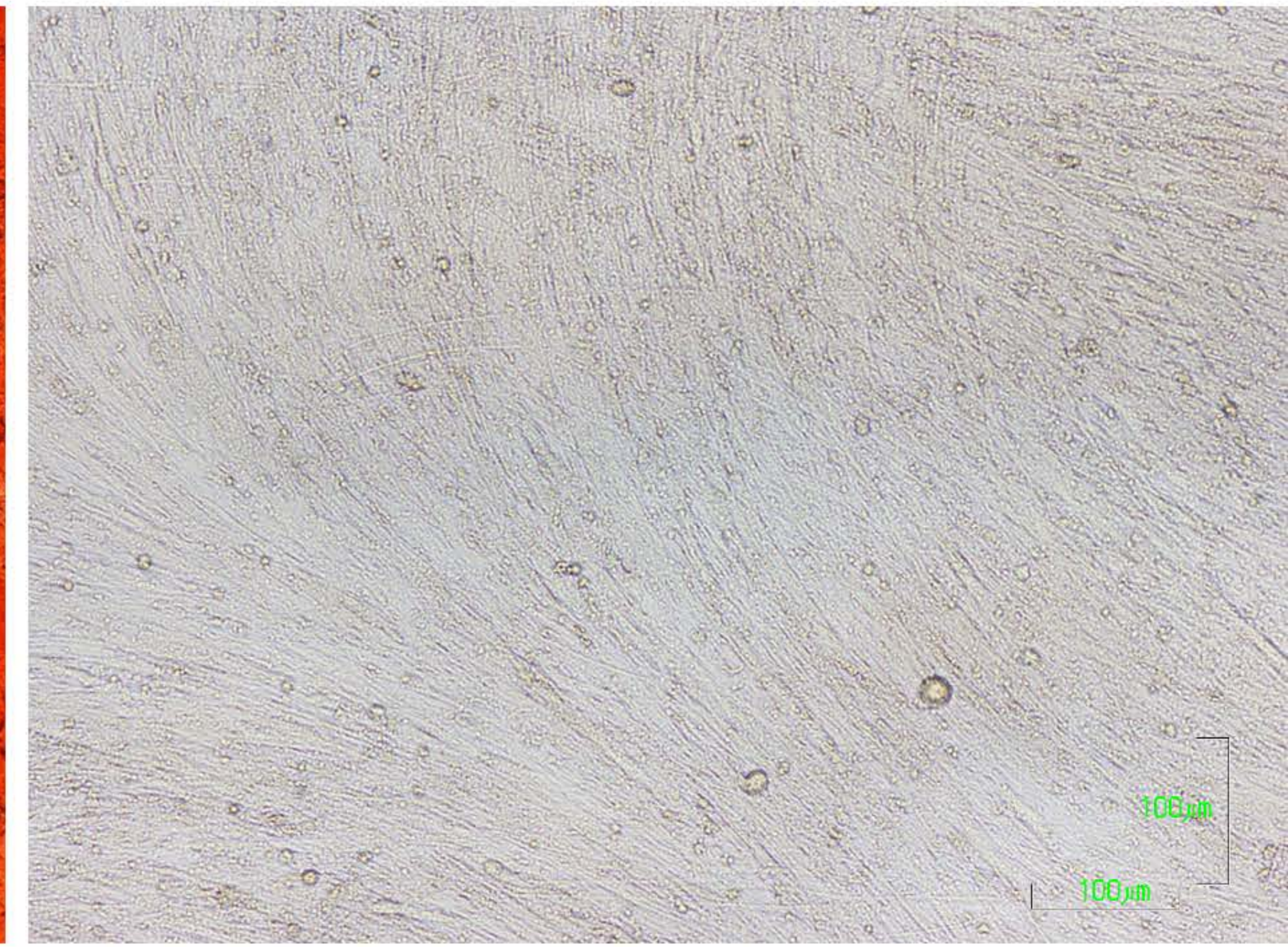

## Donor 4

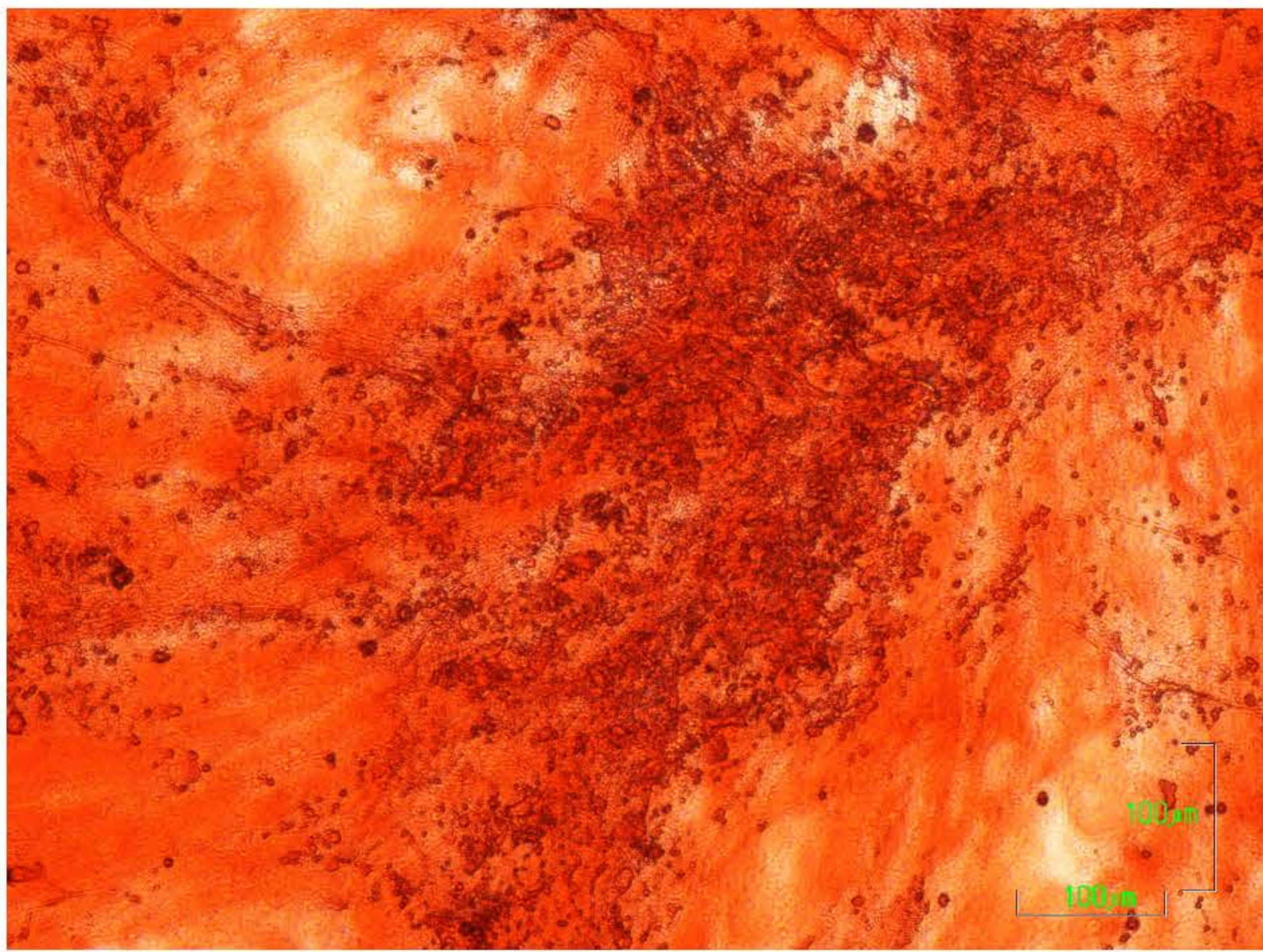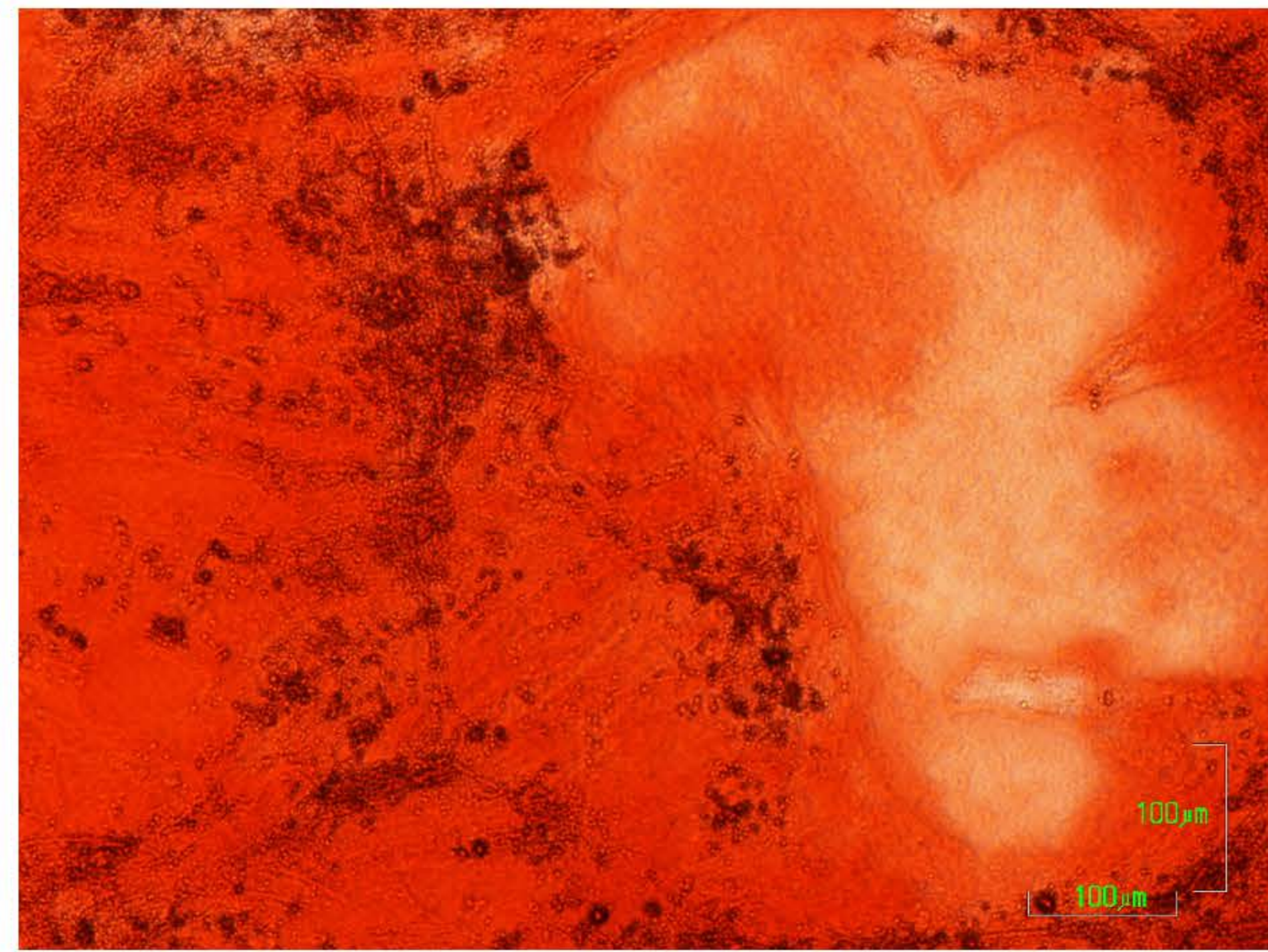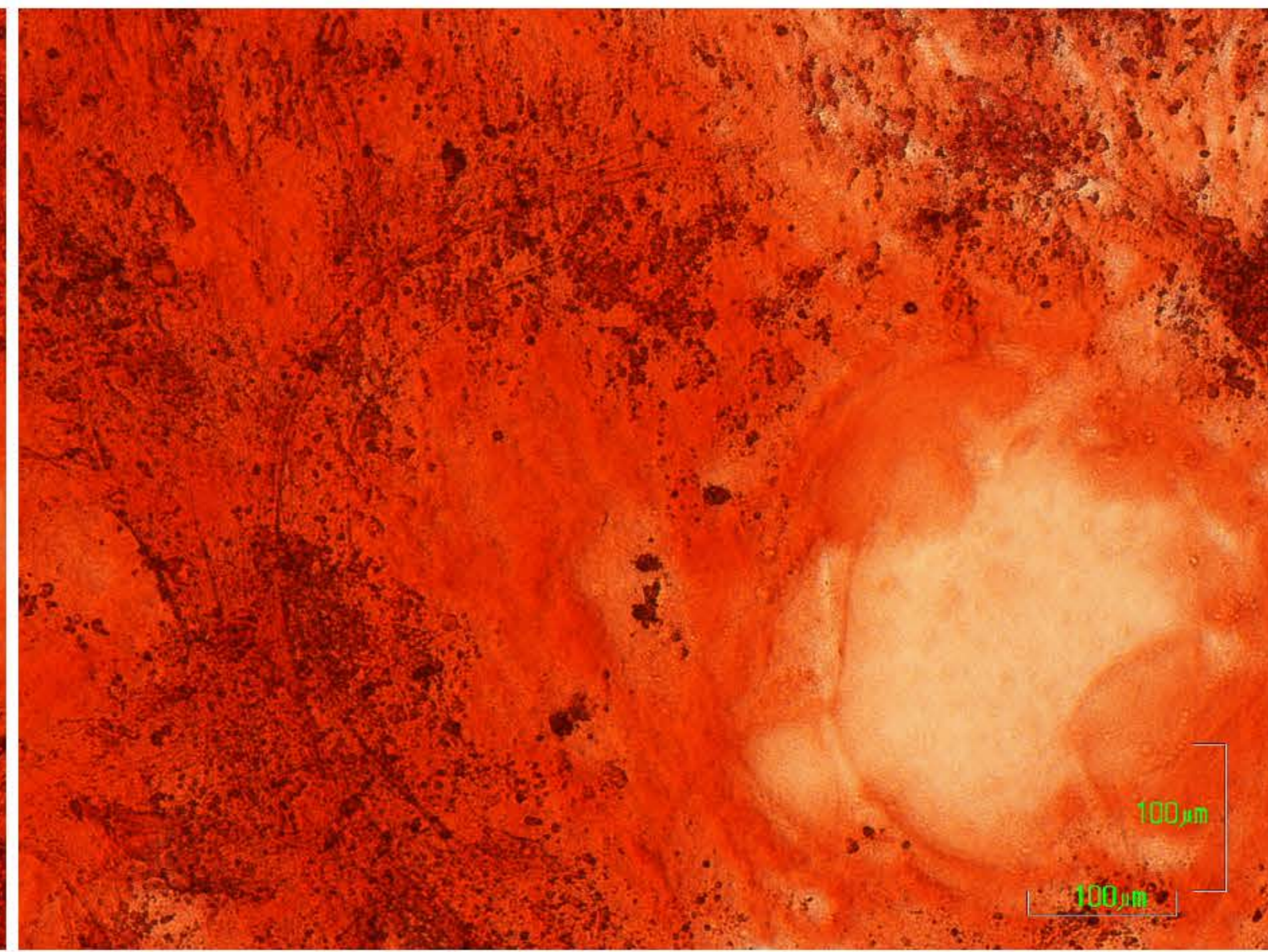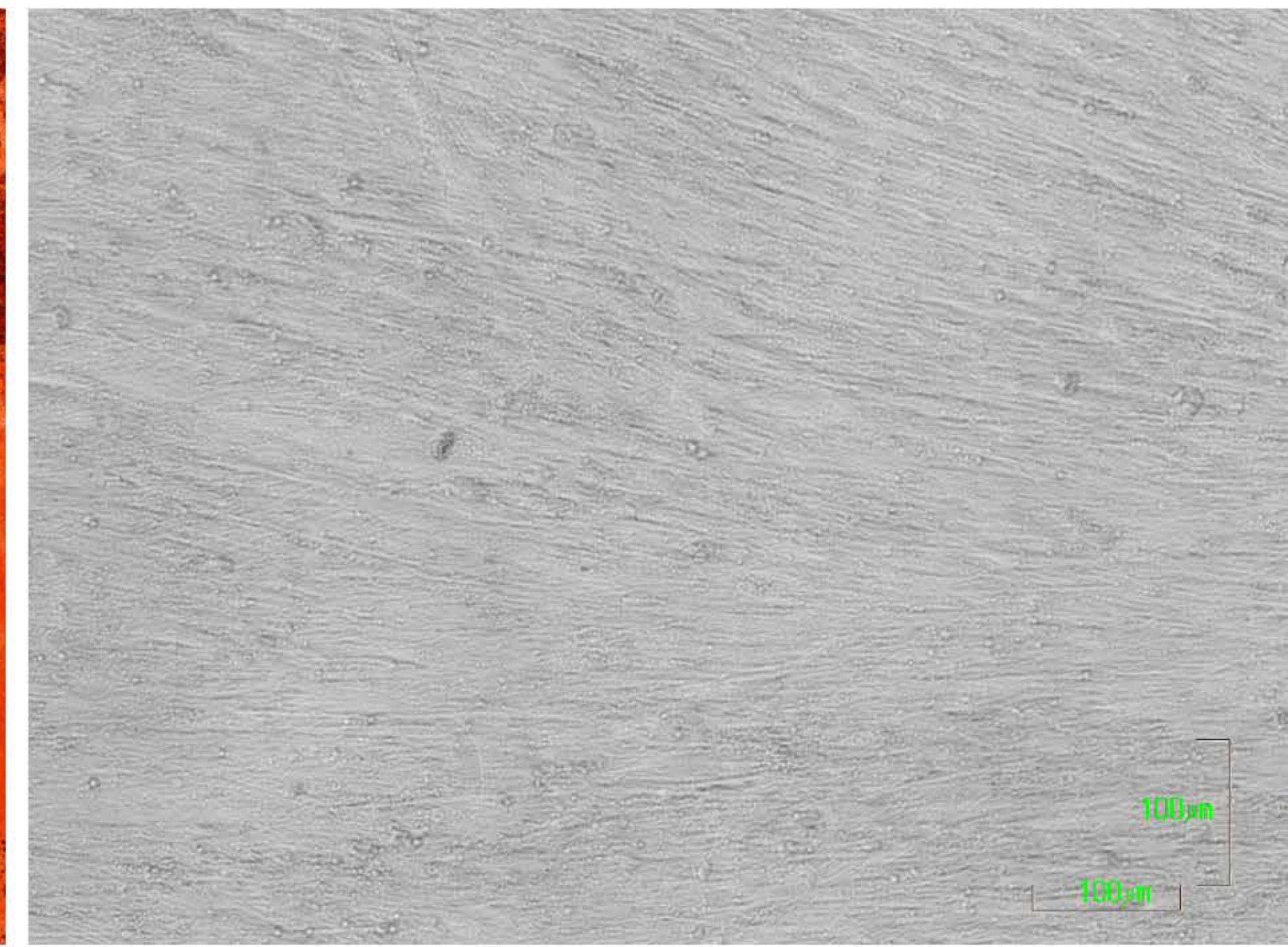

## Donor 5

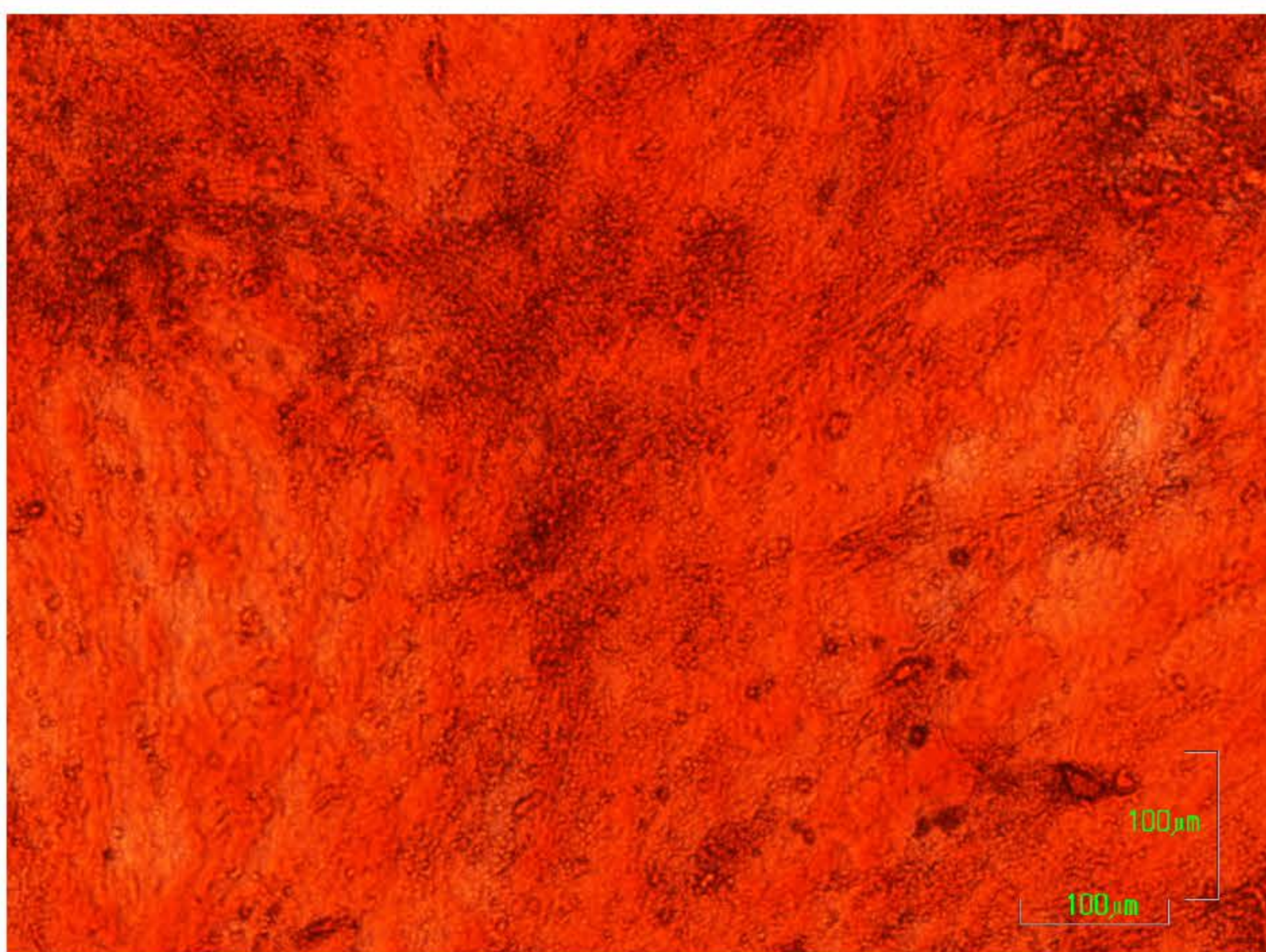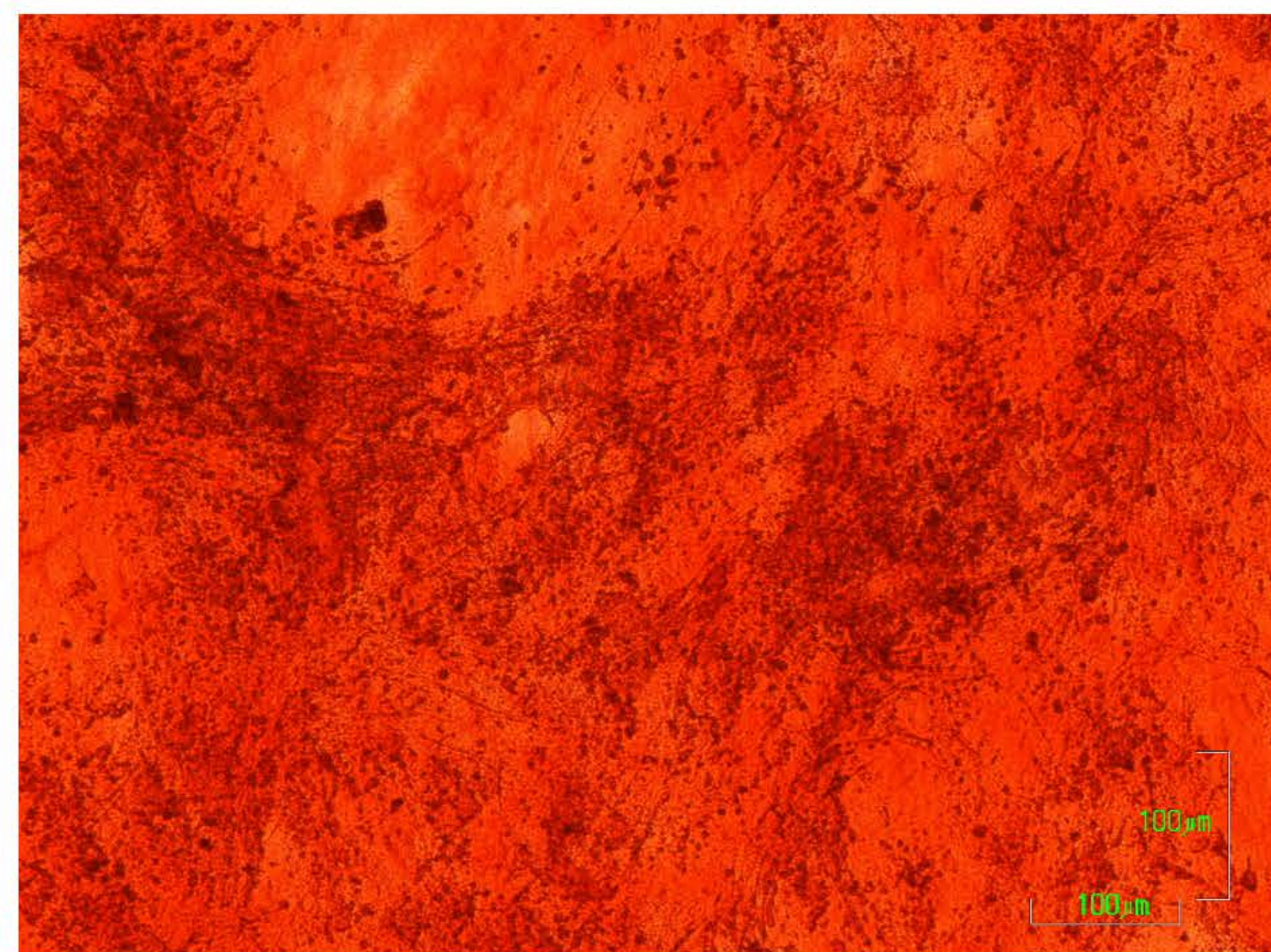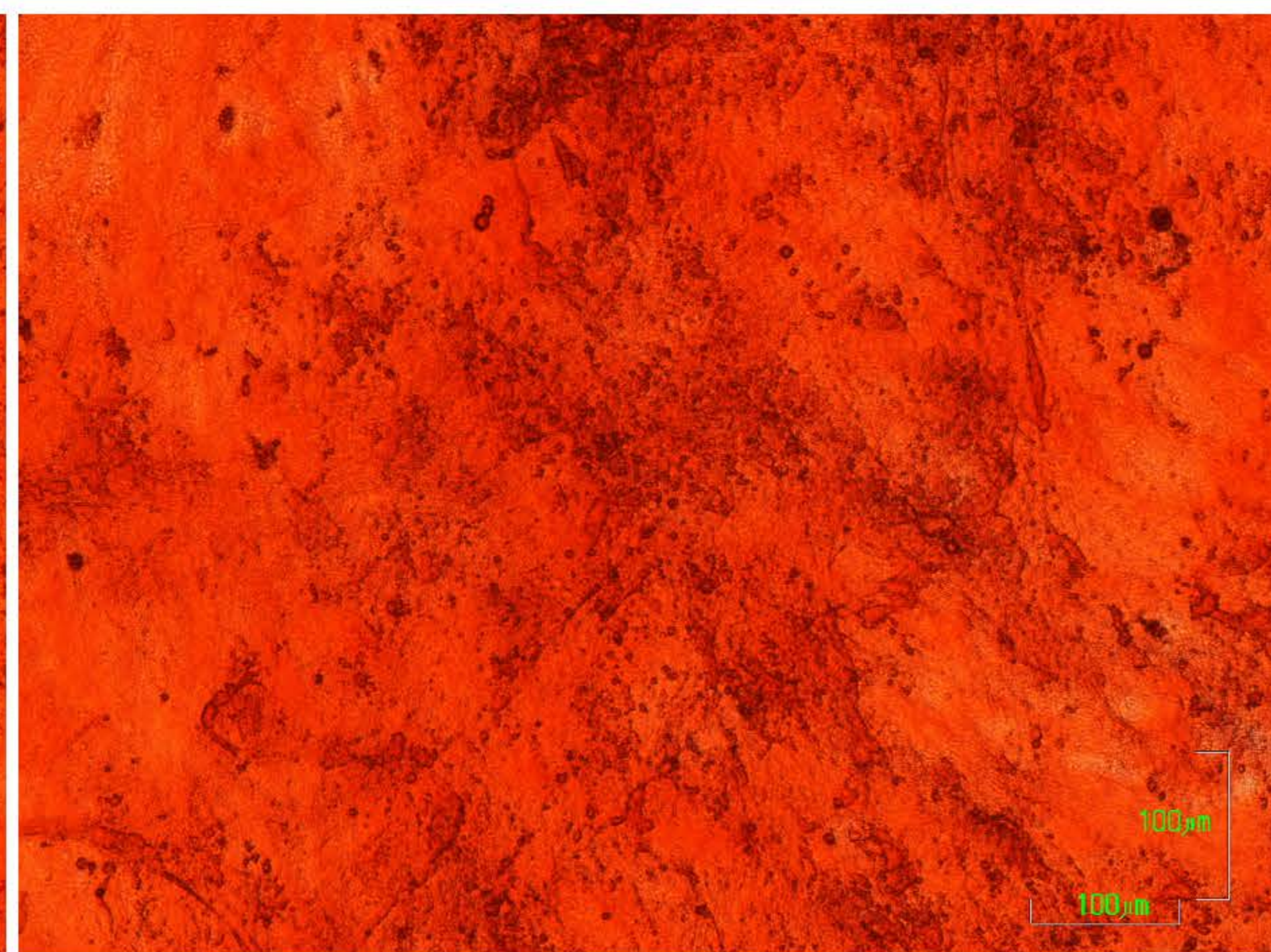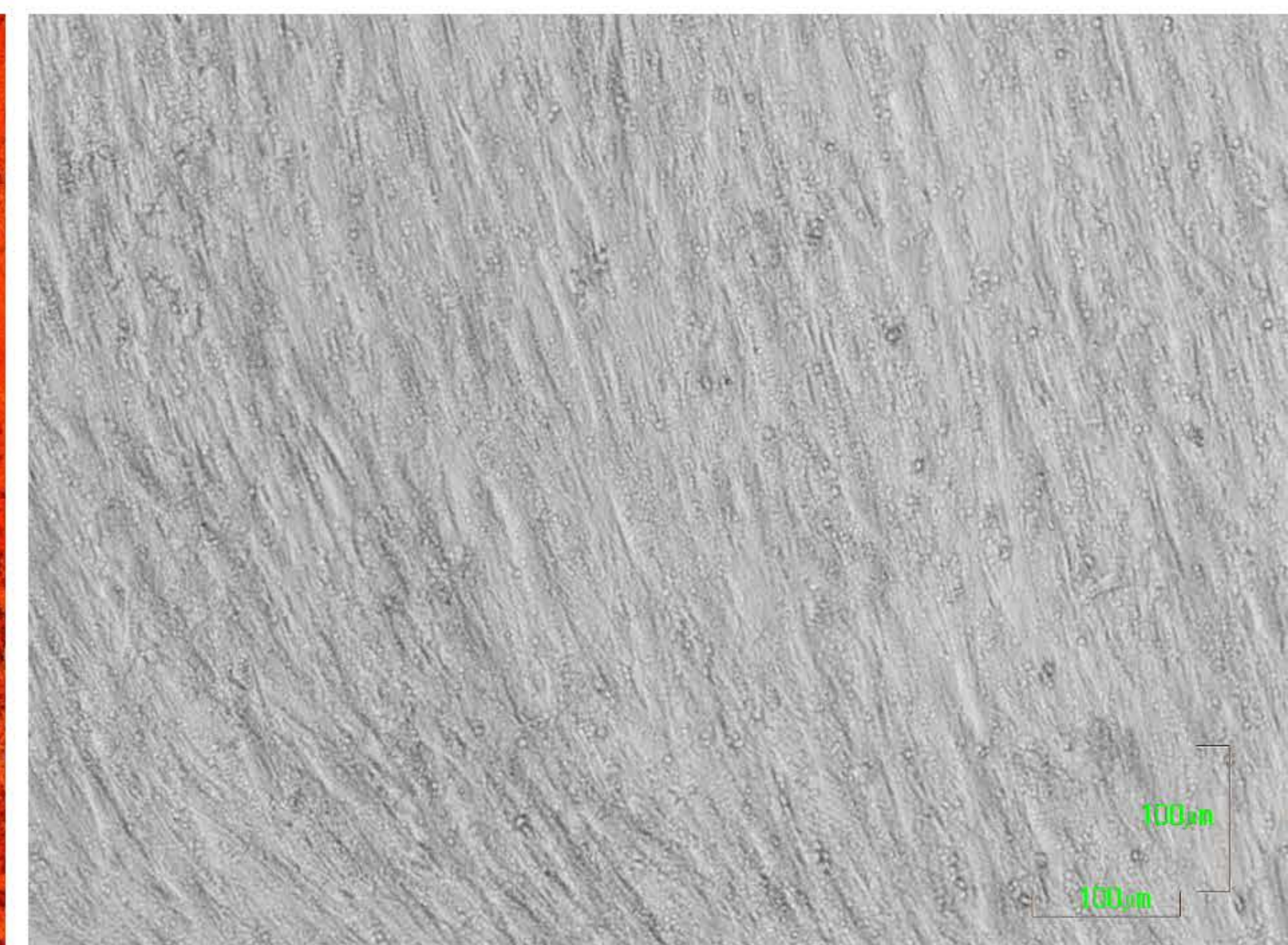

# Donor 11

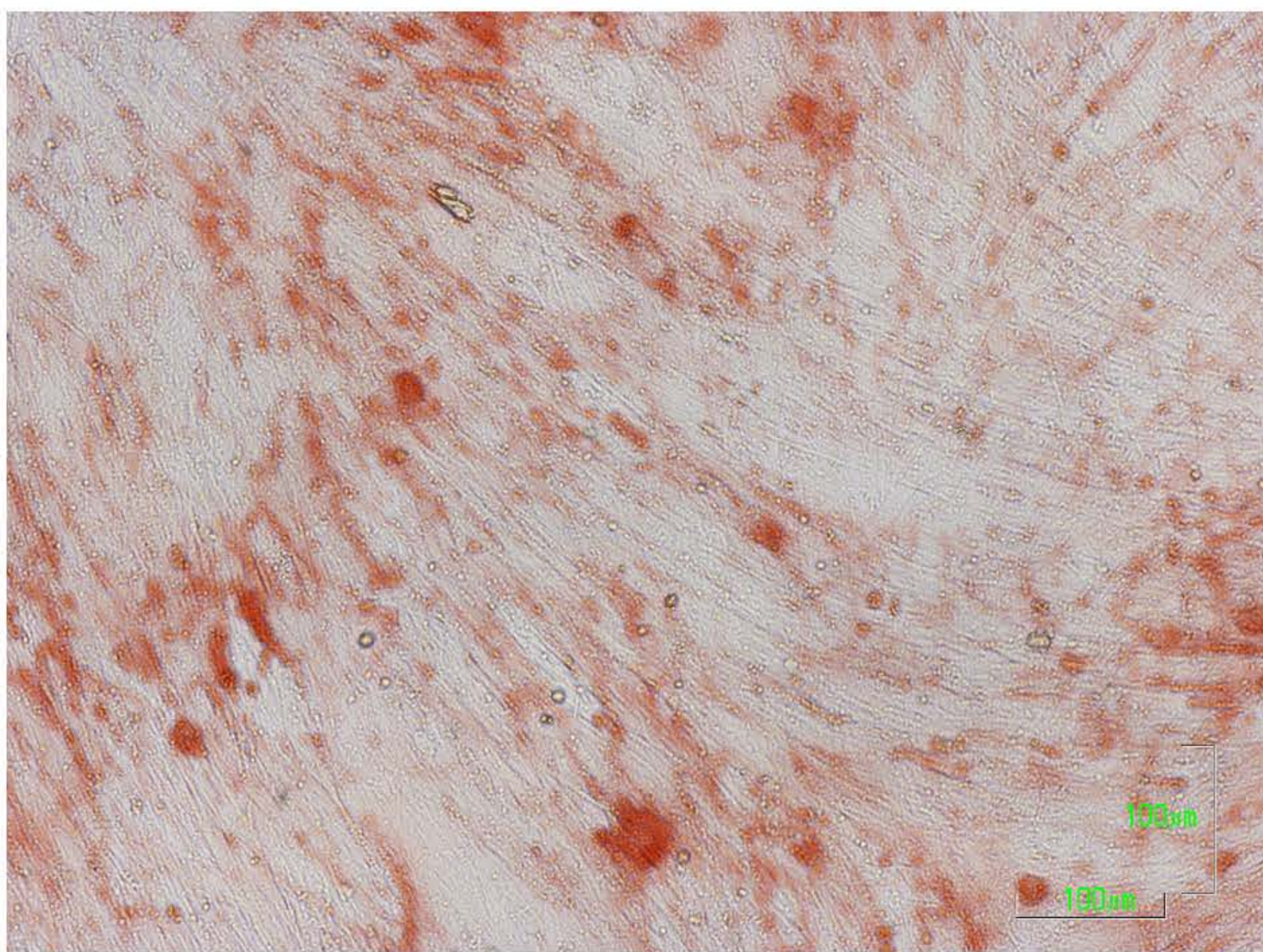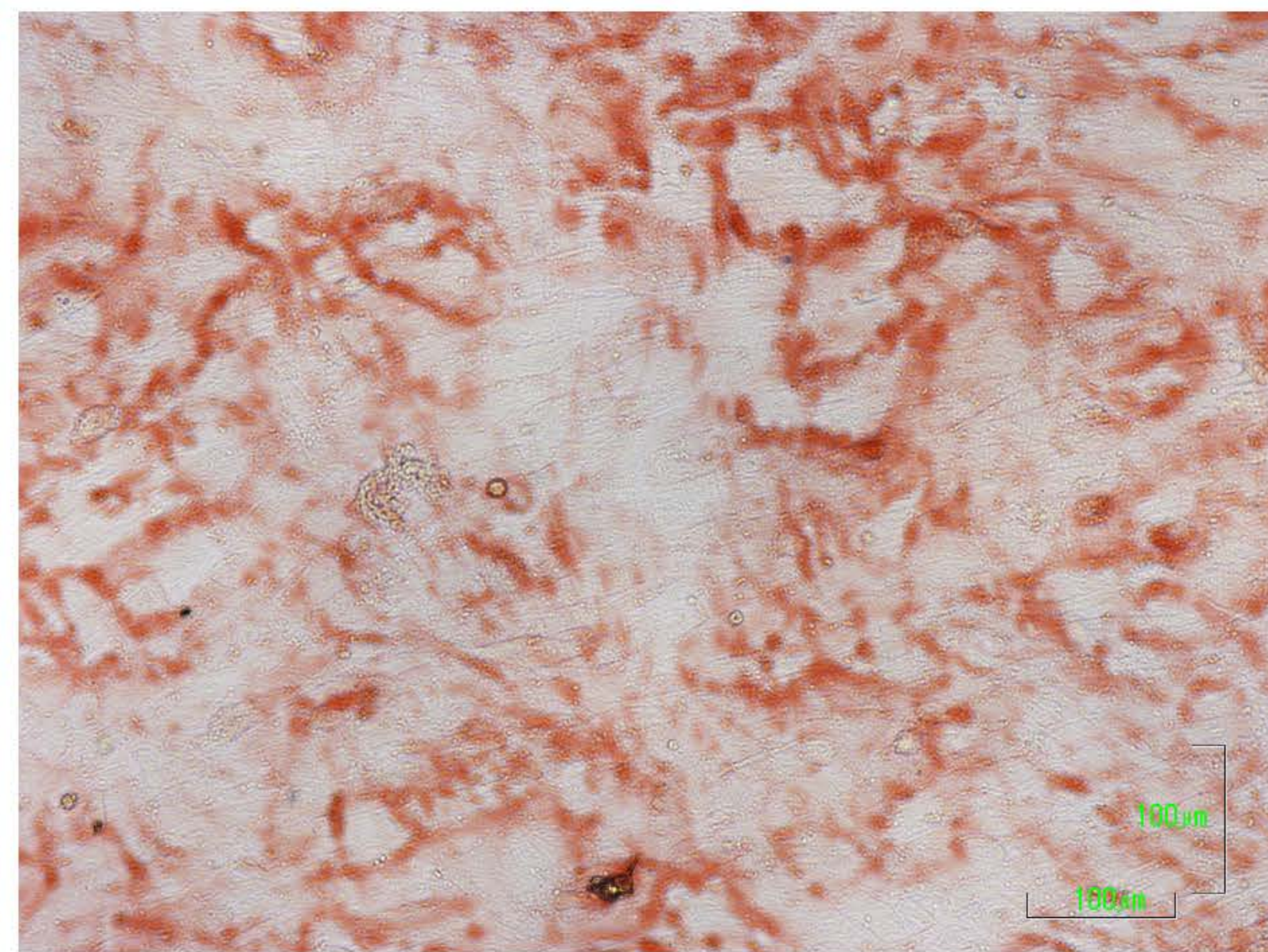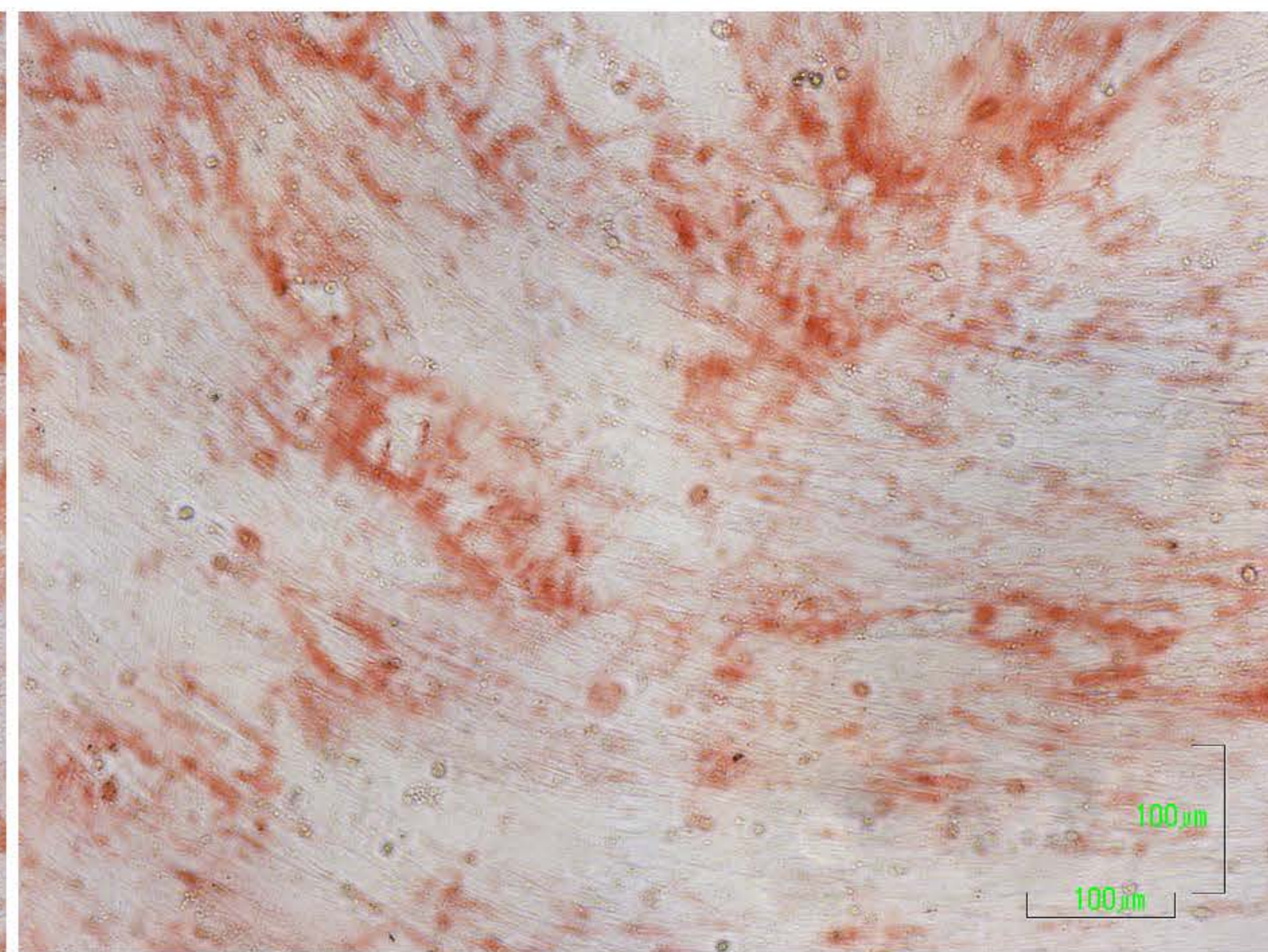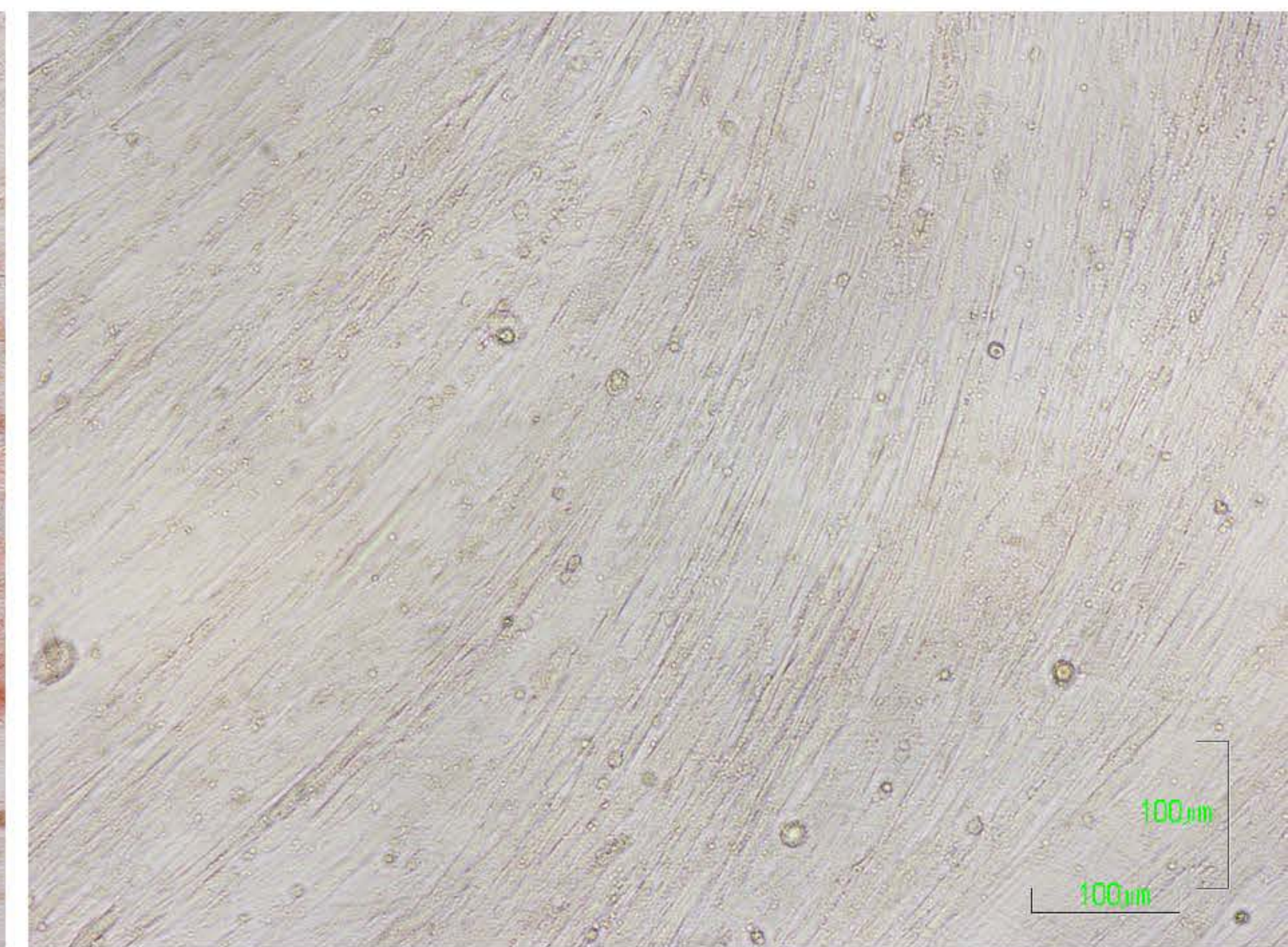

Donor 12

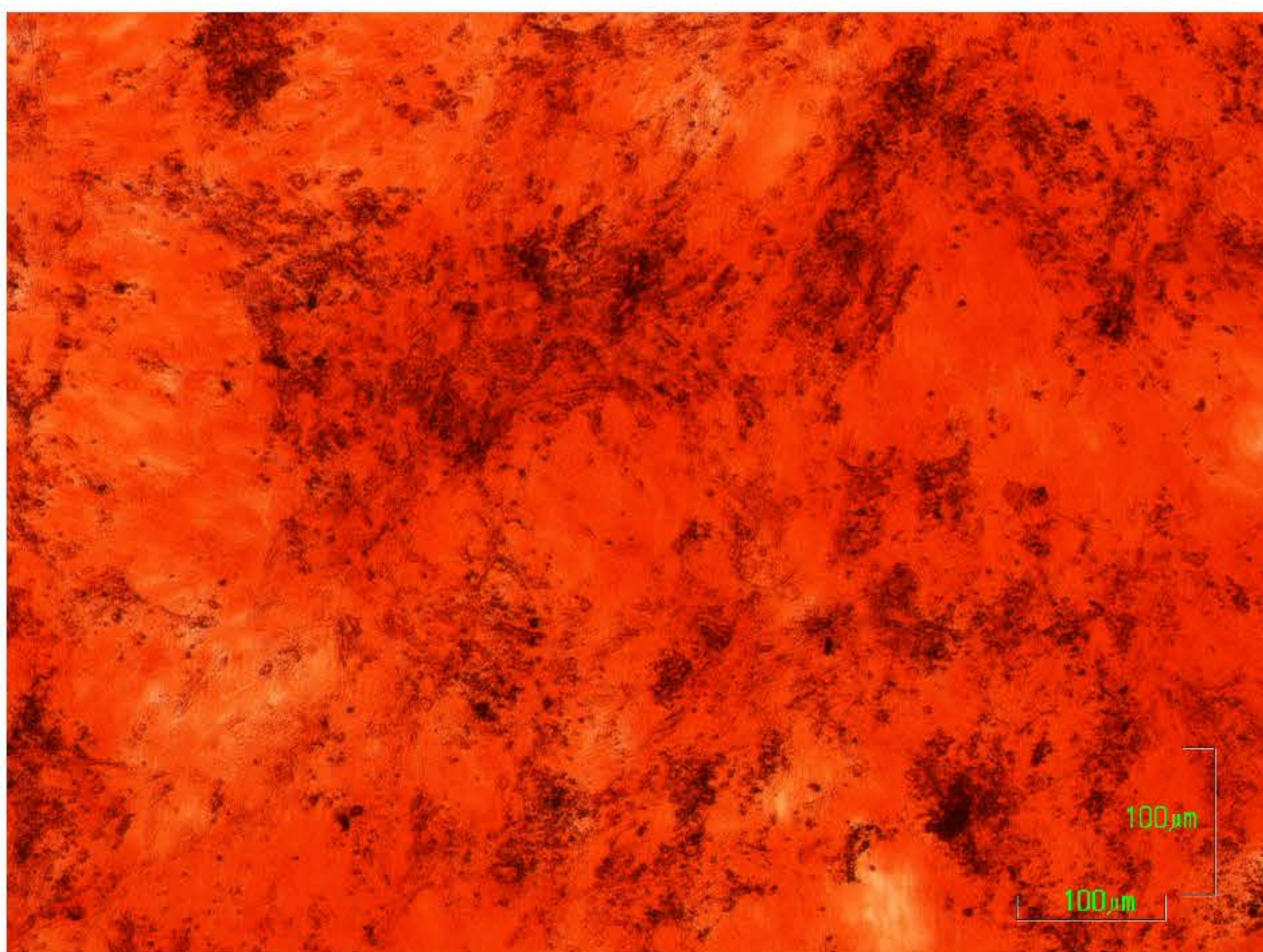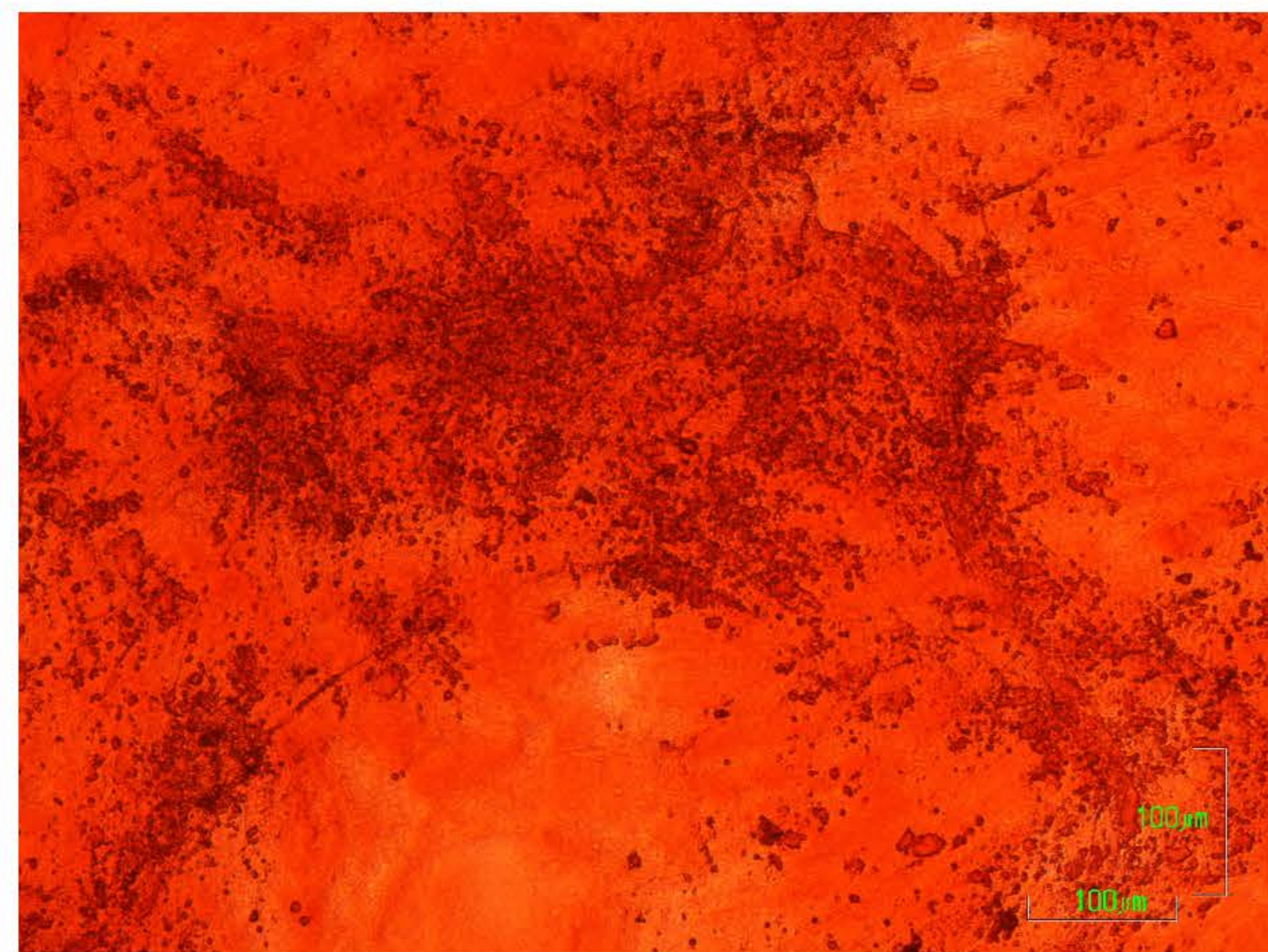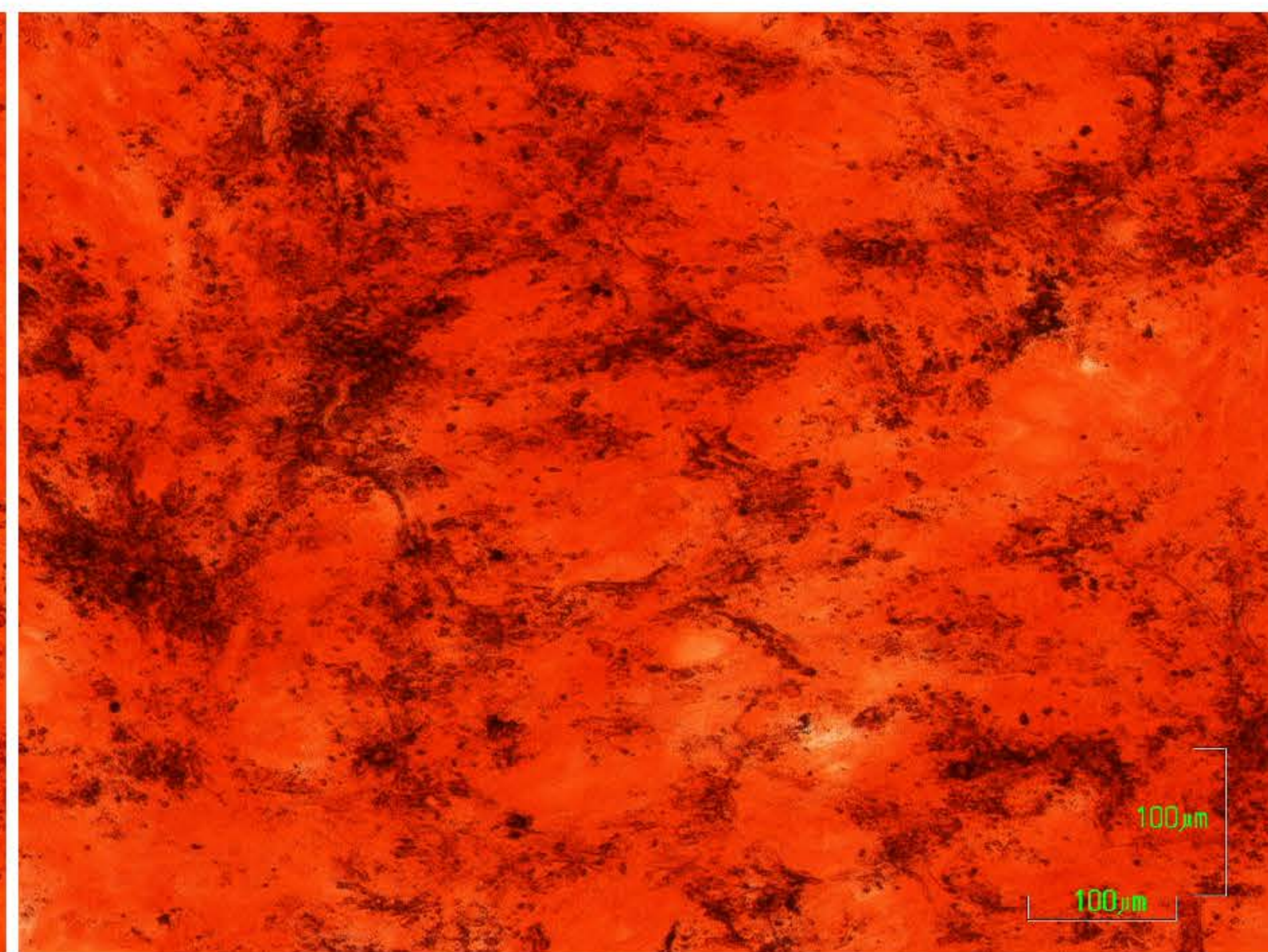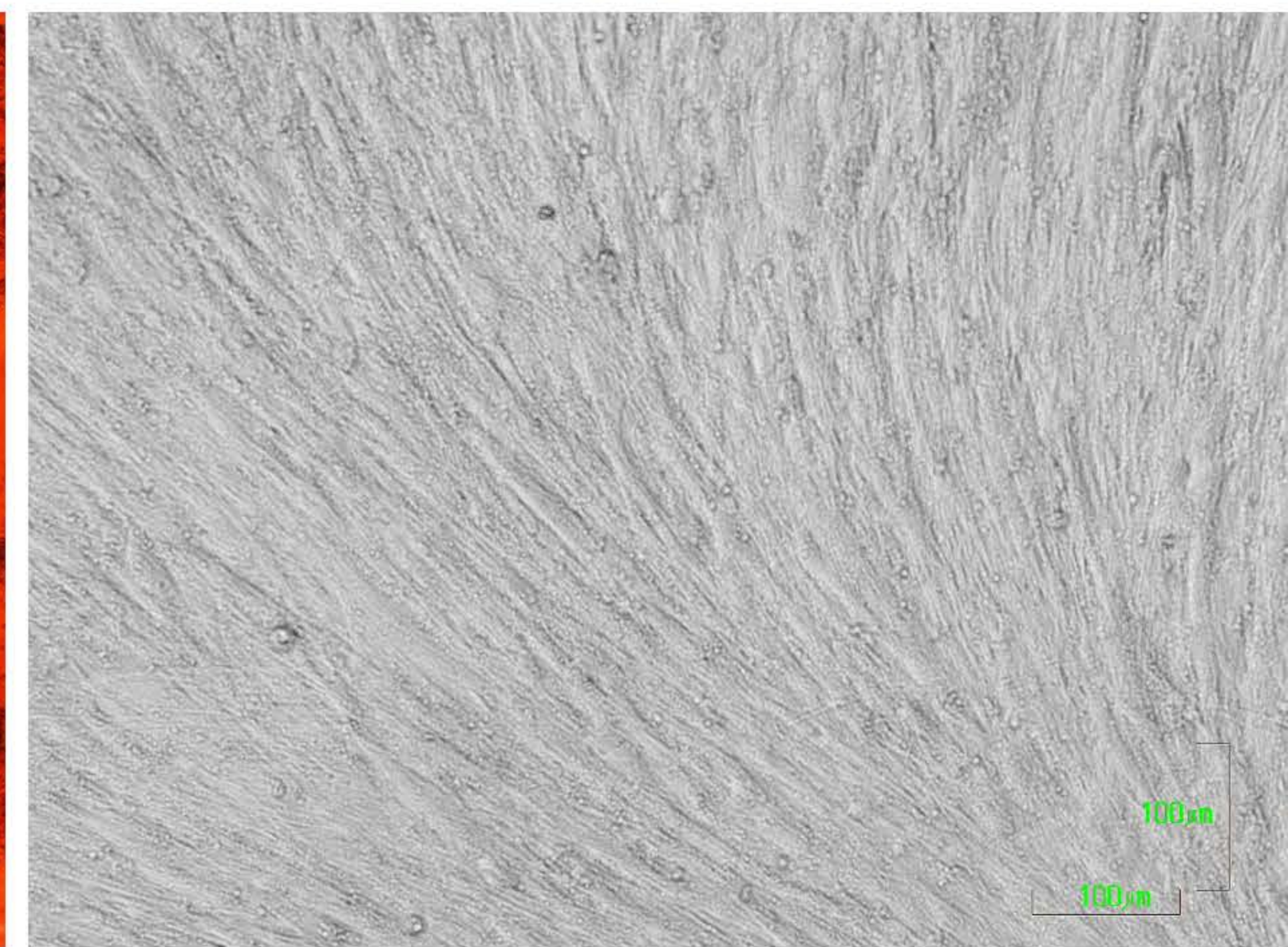

Supplement: Additional file 5: Figure S4. — Alizarin red staining of calcium nodules after osteogenic induction of hMSC isolated under varying culture condition from different donors. No differences were observed between the culture conditions, though differences between the donors were identified. Donor 2 and 11 showed less calcium nodules formation than the rest of the donors. All the controls stained negative for calcium nodules formation. Values are represented as mean ± standard deviation of at least three independent experiments (n = 3). (PDF 2096 kb) [file 12896_2016_318_MOESM5_ESM.pdf]

# Surface marker expression

CD90

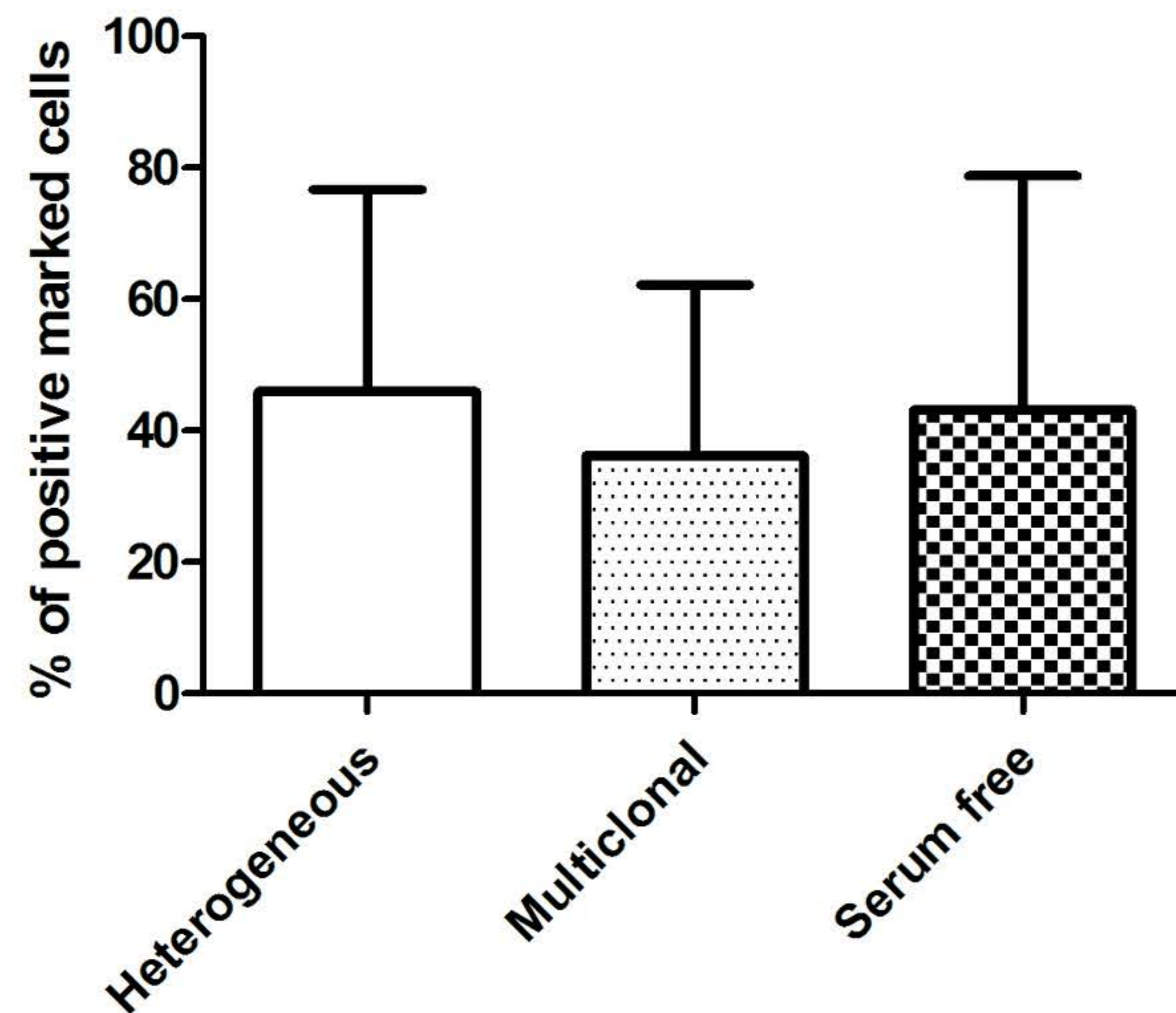

CD105

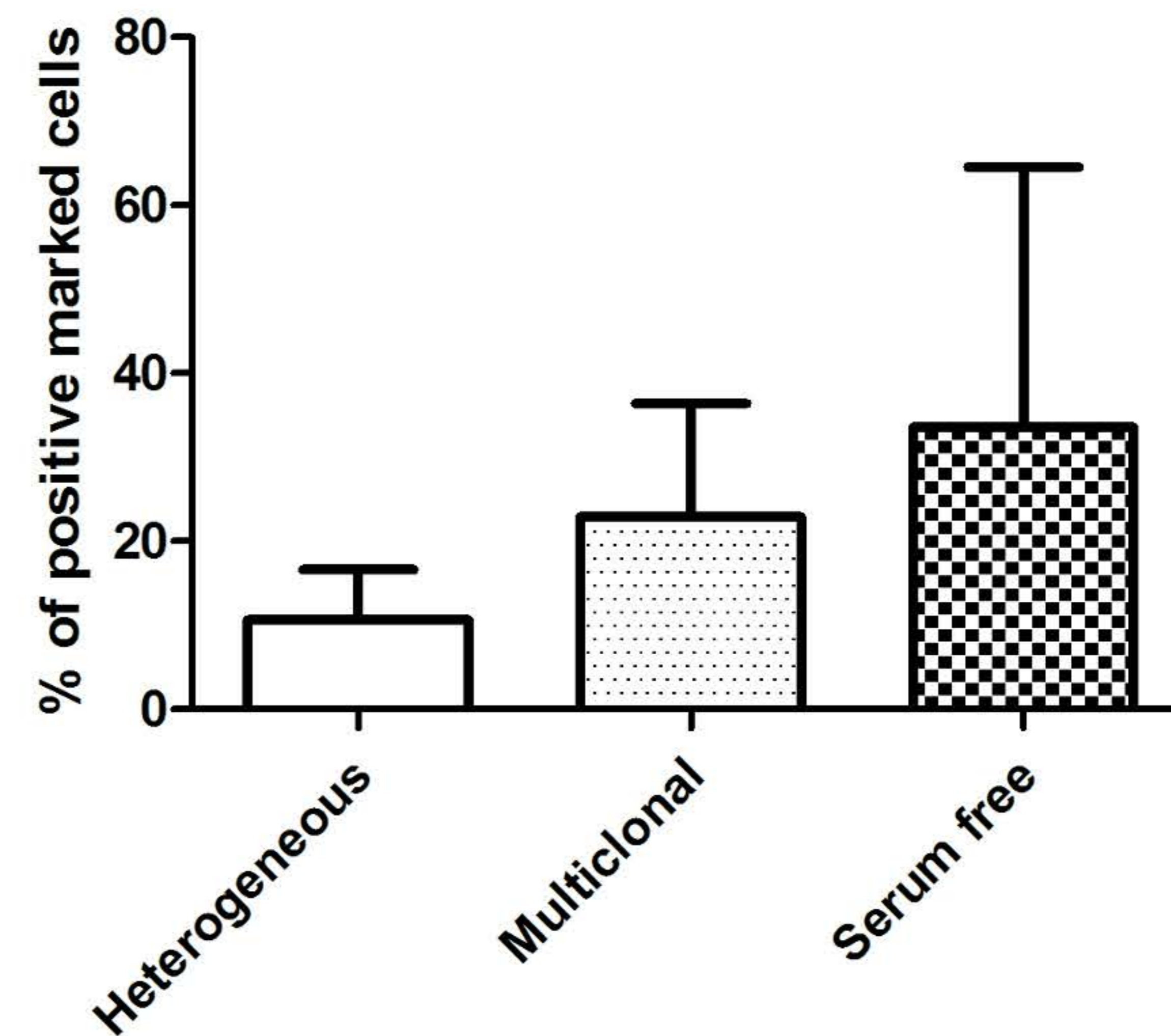

CD73

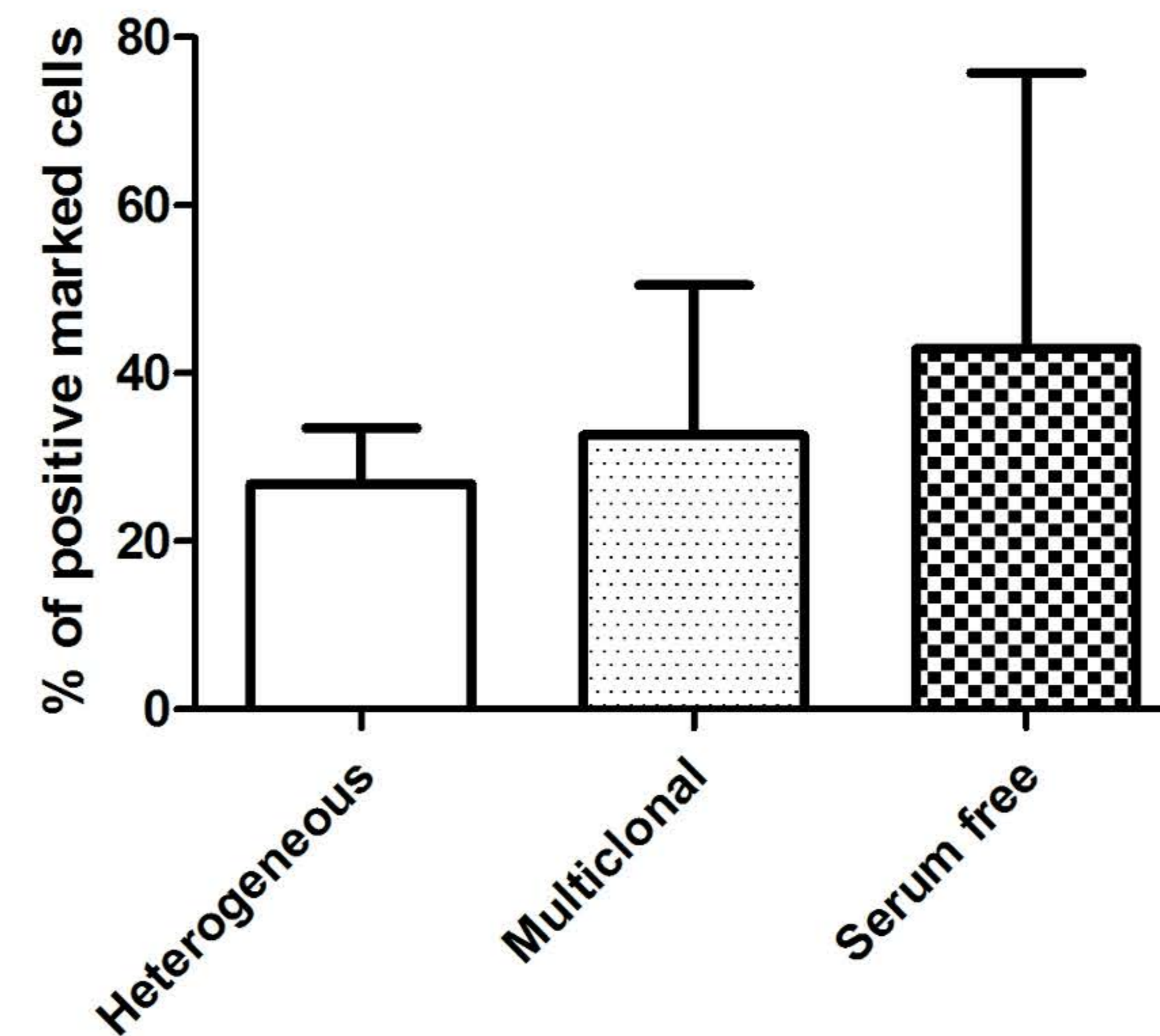

CD146

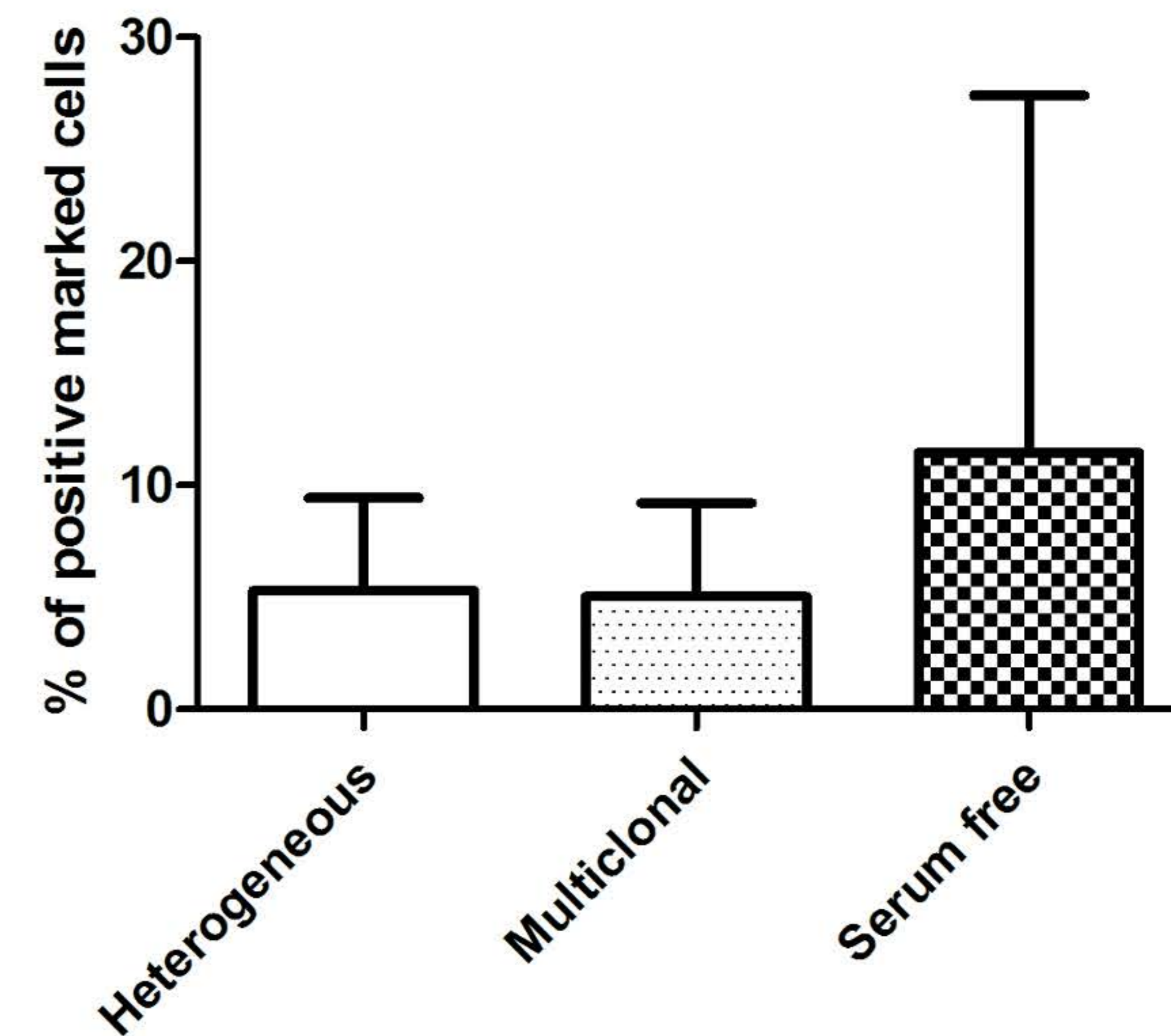

Supplement: Additional file 6: Figure S5. — Surface marker expression (in percentage) of the varying culture conditions represented as a bar plot. Each bar stands for the average over the percentage of surface markers obtained from three donors. Selected sets of cell surface markers expressed positive on hMSC. All the other investigated sets were expressed negative for both conditions, therefore not shown. Not statistically significant differences were found between the three conditions. (PDF 210 kb) [file 12896_2016_318_MOESM6_ESM.pdf]
