# Supplementary material for: Means of enhancing bone fracture healing: optimal cell source, isolation methods and acoustic stimulation
Source: BMC Biotechnol. 2016 Dec 12;16:89. doi: 10.1186/s12896-016-0318-1 (PMC5154008; doi:10.1186/s12896-016-0318-1)
Supplement: Additional file 3: Figure S2. — Biological characterization of isolated hMSCs from acoustically stimulated BM at 300 Hz for 5 min at different volumes, 11.5, 10, 8, 6 and 5 ml. The results are presented as the fold change over the non-stimulated bone marrow (baseline). (A) Graphic representation of the bone marrow volumes, donor dependent. (B) Proliferation of hMSCs calculated as PD/day from P1 to P2, donor and volume dependent. (C) CFU potential of hMSCs, donor and volume dependent. (D) ECM production, quantification of nodule size area in mm2, donor and volume dependent. (E) Osteogenic potential calculated as percentage of ALP positive colonies within the CFUs, donor and volume dependent. (F) Adipogenic potential, quantification of Oil red O staining relative to 100% Oil red O staining solution, donor and volume dependent. Values are represented as mean ± standard deviation of at least three independent experiments (n ≥ 3). Statistically significant differences were found with ***p < 0.001, **p < 0.01 and *p < 0.05. (PDF 694 kb) [file 12896_2016_318_MOESM3_ESM.pdf]

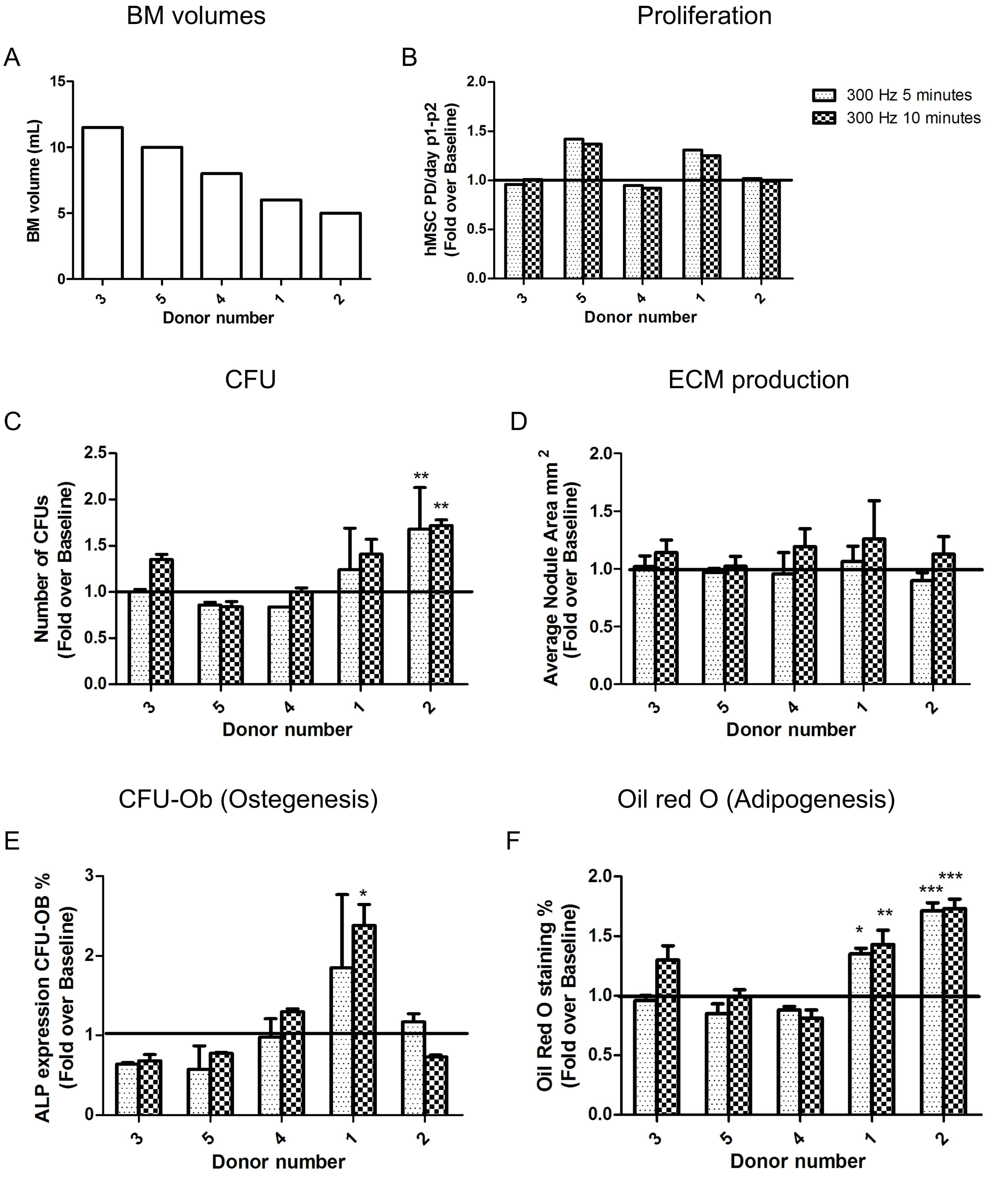

BM volumes
A
BM volume (mL)
15
10
5
0
3
5
4
1
2
Donor number
Proliferation
B
hMSC PD/day p1-p2 (Fold over Baseline)
2.0
1.5
1.0
0.5
0.0
300 Hz 5 minutes
300 Hz 10 minutes
Donor number
CFU
C
Number of CFUs (Fold over Baseline)
2.5
**
**
Donor number
ECM production
D
Average Nodule Area mm 2 (Fold over Baseline)
Donor number
CFU-Ob (Ostegenesis)
E
ALP expression CFU-OB % (Fold over Baseline)
3
2
1
0
*
Donor number
Oil red O (Adipogenesis)
F
Oil Red O staining % (Fold over Baseline)
*
**
***
***
Donor number
